# Lead-free Cs_2_Ag_1−__x_Na_x_In_1 − y_Bi_y_Cl_6_ perovskite films with broad warm-yellow emission for lighting applications

**DOI:** 10.1038/s41598-024-65492-5

**Published:** 2024-06-26

**Authors:** Haiyan Wang, Jin Chen, Yu Sun, Fengchao Wang, Jing Yang, Canyun Zhang, Jinfang Kong, Lan Li

**Affiliations:** College of Sciences, Shanghai Institute of Technology, 100 Haiquan Road, Shanghai, 201418 China

**Keywords:** Lead-free, Double perovskite, Cs_2_AgInCl_6_, Warm-yellow, Blade coating, Materials for optics, Optical materials and structures, Fluorescence spectroscopy

## Abstract

Lead-free halide double perovskite Cs_2_AgInCl_6_ has been extensively studied in recent years due to the lead toxicity and poor stability of common lead halide perovskites. In this study, sodium (Na^+^) and bismuth (Bi^3+^) doped into Cs_2_AgInCl_6_ double perovskite, then Cs_2_Ag_1−__x_Na_x_In_1 − y_Bi_y_Cl_6_ films with broadband warm-yellow emissions were achieved by the blade coating method. Herein, Na and Bi content were changed as variables at a series of parameter optimization experiments, respectively. In the Cs_2_Ag_1−__x_Na_x_In_1 − y_Bi_y_Cl_6_ systems, Na^+^ broke the parity-forbidden transition of Cs_2_AgInCl_6_, and Bi^3+^ suppressed non-radiative recombination. The partial replacement of Ag^+^ with Na^+^ ions and doping with Bi^3+^ cations were crucial for increasing the intensity of the PL emission. The experimental results showed that the photoluminescence quantum yield of the Cs_2_Ag_0.4_Na_0.6_In_0.8_Bi_0.2_Cl_6_ film was 66.38%, which was the highest data among all samples. It demonstrated remarkable stability under heat and ultraviolet conditions. After five thermal cycles, the PL intensity of the Cs_2_Ag_0.4_Na_0.6_In_0.8_Bi_0.2_Cl_6_ film is only reduced to approximately 5.7% of the initial value. After 720 h continuous ultraviolet irradiation, there occurred 31.9% emission decay of the film.

## Introduction

Lead halide perovskites have been considered as polular materials for photovoltaic and optoelectronic applications. They have a universal formula of ABX_3_ structure (A is Cs^+^, MA^+^ or FA^+^, B is Pb^2+^, and X is Cl^−^, Br^−^ or I^−^)^1–3^. However, because of their high toxicity and poor stability, the environmental-friendly lead-free halide perovskites have drawn more attention in the field. Moreover, the remarkable characteristics, including tunable optical bandgap, strong stability, easy solution processability, and good light-harvesting capability^4–8^, make these candidates feature promising potential in optoelectronic applications (such as photovoltaic^9^, lighting^10^, laser^11^, and imaging^12^). As reported by early studies, the low-toxic Sn^2+^ and Ge^2+^ have been usually used for substituting the heavy mental cations Pb^2+^ due to Sn^2+^ and Ge^2+^ are the elements in the same group with identical lone pairs as lead^13–17^. Unfortunately, Sn^2+^ and Ge^2+^ are easily oxidized to + 4 state to destruct material structure, which also leads a poor stability in the ambient atmosphere. Thus, a significant decrease in the photoelectric performance of perovskite devices is presented^5,18–20^. Furthermore, there is a discussion that Sn may be more harmful to humans than Pb when Sn is dispersed in the environment as reported by some investigations^14^. Methods that replace Pb^2+^ with Bi^3+^, Sb^3+^, or Tl^+^ may cause the charge imbalance and structure deterioration of perovskite materials. Further, Tl is highly toxic. Thus, it’s essential to find perovskites with good stability, low toxicity, and high efficiency.

In this regard, researchers have turned their eyes to the field of lead-free double halide perovskites (LFHDPs). Notably, A_2_B(I)B(III)X_6_ structure LFHDPs have been regarded as stable and environmental-friendly alternatives to ABX_3_ lead-based perovskites since 2016^21–24^. Here, B(I)-site element is a monovalent cation, such as Na^+^, K^+^, Li^+^, Tl^+^, In^+^, Cu^+^, Ag^+^, Ru^+^, and Au^+^. B(III)-site element is a trivalent cation, such as Sb^3+^, Bi^3+^, In^3+^, Al^3+^, Ln^3+^ Ga^3+^, Fe^3+^^25–27^. Wherein, Cs_2_B(I)BiX_6_ and Cs_2_B(I)SbX_6_ (B(I) = Ag, Cu, Na) are indirect bandgap materials^21,26,28^. Through first-principles calculation and experiments, Cs_2_AgInCl_6_ has received plenty of researchers’ concern and has been viewed as a promising candidate in light-emitting devices (LEDs)^29,30^, photodetectors ^31^ because of its direct bandgap and creditable properties^32–34^. Unfortunately, Cs_2_AgInCl_6_ displays a low PLQY (~ 1.6 ± 1%)^35^ since the parity-forbidden optical transitions induced from the inversion symmetry^36,37^. Moreover, Cs_2_AgInCl_6_ has characteristics of low electron dimension, big bandgap, large effective mass of charge carriers, and detrimental defects^36,38,39^. To address these difficulties, significant efforts have been invested in sodium (Na^+^) alloying and bismuth (Bi^3+^) doping into the Cs_2_AgInCl_6_ materials^40–42^. Luo et al.^29^ made an important breakthrough with this method for the first time. They mixed CsCl, NaCl, AgCl and InCl_3_ precursors into an HCl solution in a hydrothermal autoclave and finally synthesized Cs_2_Ag_x_Na_1 − x_InCl_6_ powder. Compared to the pure Cs_2_AgInCl_6_ and Cs_2_NaInCl_6_, it was found that the Cs_2_Ag_x_Na_1 − x_InCl_6_ sample brought an increase in PL emission by three orders of magnitude. With 0.04% Bi doping, Cs_2_Ag_0.6_Na_0.4_InCl_6_ could emit 86 ± 5% quantum efficiency warm-white light. Subsequently, Yang and his colleagues reported the first direct band gap Cs_2_AgIn_x_Bi_1 − x_Cl_6_ (x = 0.75 and 0.9) nanocrystals (NCs)^43^. The Cs_2_AgIn_x_Bi_1 − x_Cl_6_ NCs exhibited > 5 times photoluminescence quantum efficiency (PLQE) compared to those observed for indirect band gap NCs (Cs_2_AgBiCl_6_). Apart from this, the Cs_2_AgIn_x_Bi_1 − x_Cl_6_ NCs exhibit orange emission (forbidden transition) and violet emission (band-to-band transition) respectively. Nevertheless, there are rare reports on the A_2_B(I)B(III)X_6_ film for light-emitting application to date.

Here, Cs_2_Ag_1−__x_Na_x_In_1 − y_Bi_y_Cl_6_ films (x = 0, 0.2, 0.4, 0.6, 0.8 and 1.0), (y = 0, y = 0.2, y = 0.4, y = 0.6, y = 0.8, y = 1.0) were directly prepared for light-emitting application by a facile blade coating strategy combined with Na^+^ and Bi^3+^ doping under open-air ambient. In current work, the intrinsic relationship between Na^+^ and Bi^3+^ doping and the properties of the fabricated thin films was investigated in detail. Furthermore, the formation mechanism of the material was also analyzed. As the results demonstrated, a highly efficient and stable warm-yellow emission was achieved. 66.38% PLQY and prominent stability under heat, and ultraviolet were tested through professional characterization and testing methods, including X-ray diffraction (XRD), scanning Electron Microscopy (SEM), photoluminescence (PL), X-ray Photoelectron Spectroscopy (XPS), time-resolved photoluminescence (TRPL), UV–vis absorption, etc. The optimal Cs_2_Ag_0.4_Na_0.6_In_0.2_Bi_0.2_Cl_6_ film fabricated in the Cs_2_Ag_1 − x_Na_x_In_1 − y_Bi_y_Cl_6_ system exhibited good stability in air ambient, with 5.7% emission decay after five thermal cycles and 31.9% emission decay after 720 h continuous ultraviolet irradiation.

## Result and discussion

### Na^+^ doping

**Figure 1:**
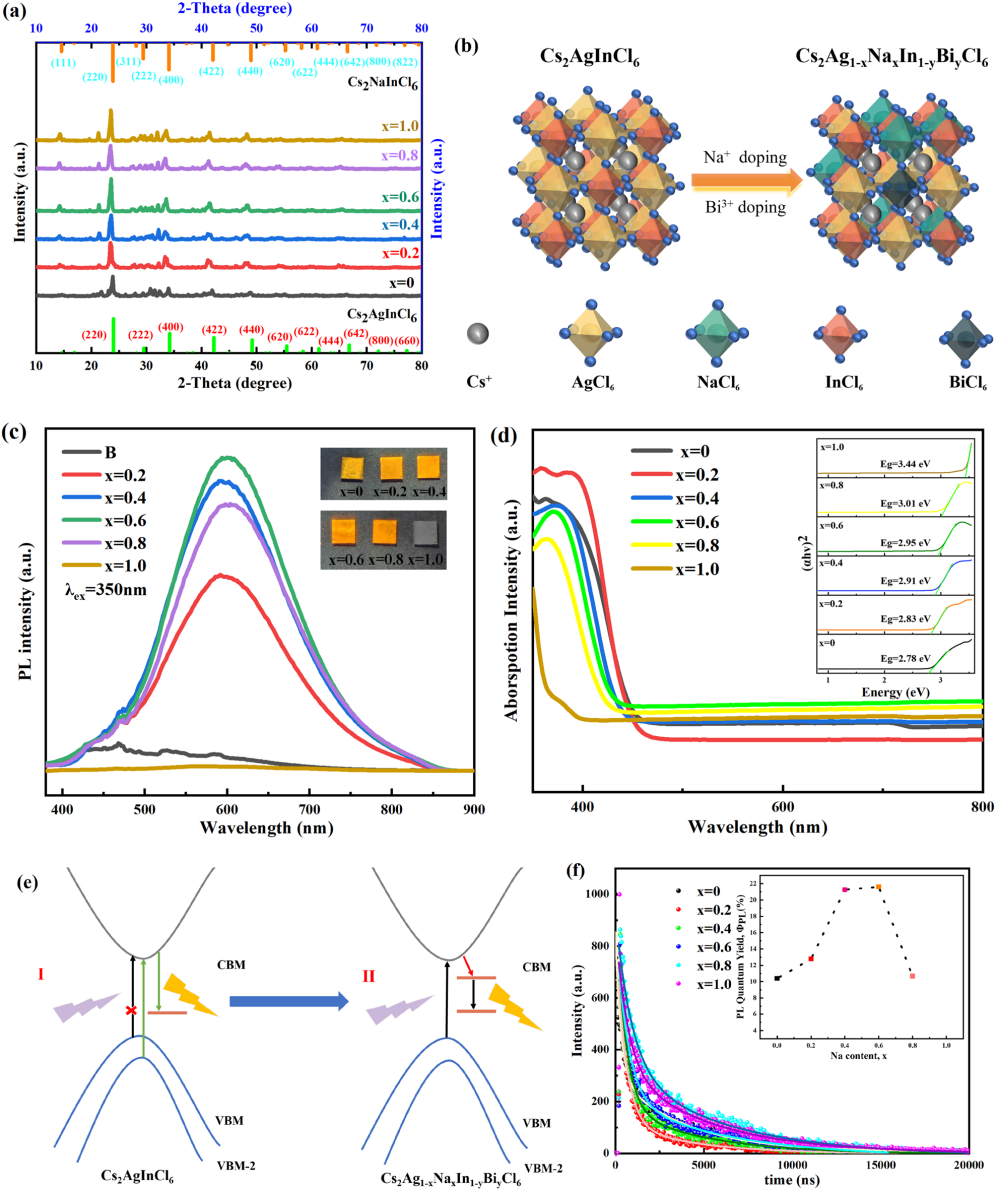
Structural characterizations and optical property of Cs_2_Ag_1 − x_Na_x_In_0.5_Bi_0.5_Cl_6_ films. (**a**) XRD patterns of Cs_2_Ag_1 − x_Na_x_In_0.5_Bi_0.5_Cl_6_ at different value of x. (**b**) Structural model of Cs_2_Ag_1 − x_Na_x_In_1 − y_Bi_y_Cl_6_. (**c**) PL spectra of Cs_2_Ag_1 − x_Na_x_In_0.5_Bi_0.5_Cl_6_ films at increasing x from x = 0 to x = 1 with 0.2 steps. The samples were all excited under 350 nm ultraviolet. The inset picture is the Cs_2_Ag_1 − x_Na_x_In_0.5_Bi_0.5_Cl_6_ films prepared by our experiment procedures under the 365 nm ultraviolet irradiation. (**d**) Optical absorption spectra of Cs_2_Ag_1 − x_Na_x_In_0.5_Bi_0.5_Cl_6_ films at increasing x from x = 0 to x = 1 with 0.2 steps, and Tauc plots (the inset picture). (**e**) Fluorescent mechanism of (I) Cs_2_AgInCl_6_ films, (II) and after Bi doping, and Na-doping films (**f**), Time-resolved PL decay curves of Cs_2_Ag_1 − x_Na_x_In_0.5_Bi_0.5_Cl_6_ measured at 590 nm, λ_exc_ = 350 nm. PLQY results are shown in the upper right corner.

In order to find the optimal stoichiometric number in this Cs_2_Ag_1-x_Na_x_In_1-y_Bi_y_Cl_6_ system, firstly, we fixed the Bi dopant amount (y = 0.5) and set Na content to a tunable figure (x ranging from 0 to 1, the step size was 0.2). Based on previous research^29,43–45^, we found that a small amount of Bi^3+^ doping is essential for the luminescence of Cs_2_AgInCl_6_. Therefore, as the intermediate value of 0 ~ 1, 0.5 was chosen as the fixed Bi doping amount to prepare Cs_2_Ag_1 − x_Na_x_In_0.5_Bi_0.5_Cl_6_ films. Figure 1a is the XRD patterns of Cs_2_Ag_1−x_Na_x_In_0.5_Bi_0.5_Cl_6_ films (x = 0, 0.2, 0.4, 0.6, 0.8, 1.0). It reveals that the XRD patterns mostly match that of the standard file of Cs_2_AgInCl_6_ (COD number 1546186, a = b = c = 10.469 Å) and Cs_2_NaInCl_6_ (COD number 4003575, a = b = c = 10.5141 Å). Moreover, the XRD plot indicates that the films are in cubic Fm-3 m DP structure. At 14.58°, it corresponded to the (111) peak of Cs_2_NaInCl_6_, which manifested that Na^+^ cations were successfully incorporated into the product, Na^+^ cations partially replaced Ag^+^ cations at the B^+^ sites of the LFHDPs structure. It can be seen the intensity of (111) peak almost continuously increases as x increases from 0 to 1, indicating an increase in the incorporation of Na^+^. The intensity of the (111) diffraction peaks is related to the Na/Ag composition through the dispersion factors of Na, Ag, and In atoms^29^. It is common to find the disorder that lies in A_2_BB’O_6_ double perovskite materials, which creates antisite defects. The appearance of the (111) peak is fundamentally attributed to the ordered arrangement of B(I) and B(III) in the Cs_2_B(I)B(III)X_6_ perovskites^29^. Therefore, it indicates that no additional disordering caused by Na, Ag alloying in Cs_2_Ag_1-x_Na_x_In_0.5_Bi_0.5_Cl_6_ films. From a structural perspective of Cs_2_AgInCl_6_ and Cs_2_NaInCl_6_, they are both LFHDPs and have a low lattice mismatch (0.3%)^29^, which creates favorable conditions for Na alloying. We also used the above-mentioned experimental methods to make Cs_2_AgInCl_6_ and Cs_2_NaInCl_6_ samples in order to figure out the component of Cs_2_Ag_1 − x_Na_x_In_1 − y_Bi_y_Cl_6_ miscellaneous peaks.

Compared with standard XRD data, Supplementary Fig. 1a shows that they fitted well at main peaks respectively, which manifests that the miscellaneous peaks did not originate from the parent Cs_2_AgInCl_6_ and Cs_2_NaInCl_6_. They may be mainly produced by impurities caused by Na^+^ and Bi^3+^ cations doping. Subsequently, we analyzed the main composition of the spurious peaks in the XRD plots, as shown in Supplementary Fig. 2a,b. It can be seen that apart from Cs_2_AgInCl_6_ and Cs_2_NaInCl_6_ diffraction peaks, the other miscellaneous peaks are mainly the peaks of AgCl and InCl. The structural model of Na alloying and Bi-doped Cs_2_AgInCl_6_ DP is shown in Fig. 1b. The cubic unit cell framework is constructed by [AgCl_6_], [NaCl_6_], [BiCl_6_] and [InCl_6_] octahedra^45^. Figure 1c is the PL spectra of Cs_2_Ag_1 − x_Na_x_In_0.5_Bi_0.5_Cl_6_ films. Compared to undoped Cs_2_AgInCl_6_, they show a broad emission spectrum. It can be obviously seen the improvement of the PL intensity from x = 0 to x = 0.6, and then began to decline from x = 0.6 to x = 1.0. The inset pictures show the Cs_2_Ag_1 − x_Na_x_In_0.5_Bi_0.5_Cl_6_ films prepared in the experiment under the 365 nm ultraviolet irradiation. Except for the sample with x = 1.0, all other samples emitted warm-yellow light. Furthermore, the optical properties of the Cs_2_Ag_1 − x_Na_x_In_0.5_Bi_0.5_Cl_6_ films were examined. In Fig. 1d we report the optical absorption spectra of these films with increasing Na content. Tauc plots are drawn based on Fig. 1d. It can be seen the six samples present a continuous increase in the bandgap from 2.78 to 3.44 eV. The causes of bandgap variations are explained later.

In order to figure out the luminescence mechanism, the internal structure of Cs_2_AgInCl_6_ before and after doping is discussed. Figure 1e depicts the mechanism of parity-forbidden transitions in Cs_2_AgInCl_6_ DPs. Inversion symmetry-induced parity-forbidden transitions can explain the phenomenon observed in the optical bandgap of Cs_2_AgInCl_6_^36^. Electrons change the parity of their spin and orbit simultaneously during the transition process, which causes energy level splitting between Ag and In, forming even and odd forbidden bands. Transitions from VBM to CBM of carriers was parity-forbidden. Simultaneously, absorption was also forbidden. From VBM-2 to CBM, carriers were parity-allowed, which is about 1.10 eV larger than the bandgap between VBM and CBM^28,42^. After Na ions doping, on the one hand, the parity prohibition transition of Cs_2_AgInCl_6_ was broken^46,47^. Radiation recombination in Cs_2_Ag_1 − x_Na_x_In_1 − y_Bi_y_Cl_6_ system was allowed due to the electron wave function changing from symmetry to asymmetry^29^. During the forbidden state, the absorption coefficient was influenced but didn’t affect the process of photoluminescence^31,36,43,45^. On the other hand, the electronic dimensionality of the system decreased by partially isolating [AgCl_6_] octahedrons. Besides, trace Bi^3+^ cations diminished defects and depressed the non-radiation recombination in Cs_2_Ag_1 − x_Na_x_In_1 − y_Bi_y_Cl_6_ system, which further enhanced their PLQYs and PL intensity^29,44,46,48^, as discussed in the later section. Many previous studies have reported that self-trapped excitons (STEs) exsit in semiconductors with localized carriers and soft lattices can exhibit broad emission^29,46,49,50^. STEs luminescence have characteristics such as wide spectrum, low absorption, and large Stokes displacement. After Na doping, STEs were spatially confined by [NaCl_6_] octahedra, resulting in enhanced orbital overlap of electron and hole^29,46^. In the excited state, STEs were generated through the Jahn–Teller distortion of [AgCl_6_] octahedrons^29,43,45,46^. In our system, efficient warm-yellow broadband emission was achieved through STEs radiation recombination as the previous literature^29,30,44,51^. Besides, the higher emission intensity of PL compared to Cs_2_AgInCl_6_ may be attributed to the trapped emission between states localized in the [BiCl_6_] and [AgCl_6_] octahedrons respectively^44^. As discussed earlier in Fig. 1d, the bandgap becomes larger. The change in the bandgap is due to the Na^+^ doping, which affects the positions of VB in the energy band. The increase of Na^+^ ions can increase the spatial overlap between CB and VB, thereby enhancing the oscillation intensity of electronic transitions^44^. However, the PL spectral position does not change significantly. This indicates that the PL does not originate from the emission of band-edge carriers but stems from STEs recombination. The photo holes are located in the Ag^+^-related internal state above the VB, while the photoelectrons are trapped in the Bi^3+^-state below the CB. The distance between these two states is almost constant, so there is no variation of the PL peak energy^44^. The schematic depiction of the energy levels involved in the photophysics of CsAg_1 − x_Na_x_In_0.5_Bi_0.5_Cl_6_ films is shown in Supplementary Fig. 3. Then we measured the PLQYs of the Cs_2_Ag_1 − x_Na_x_In_0.5_Bi_0.5_Cl_6_ films. The testing method of PLQY is detailed in the Supplementary information. Supplementary Fig. 4 shows a test plot of one of the Cs_2_Ag_1 − x_Na_x_In_1 − y_Bi_y_Cl_6_ films. The inset represents a magnified view of the emission spectrum. From Fig. 1f, when x = 0.6, Cs_2_Ag_0.4_Na_0.6_In_0.5_Bi_0.5_Cl_6_ film shows the highest value of PLQY (21.6%). The testing method and diagram for PLQY are included in the Supplementary information. As the Na content continues to increase, conversely, PLQY decreases. This is consistent with the PL results. An important factor may be attributed to the increase of the non-radiation recombination caused by electron–phonon coupling in the system^29,44^. When x = 0, x = 1.0, non-radiation recombination occupied a dominant stage, with fluorescence quenching. To understand the recombination dynamics of the exciton, time-resolved PL measurements are performed as shown in Fig. 1f. All decay curves are fitted well by a biexponential decay function, and the fitting parameters are listed in Supplementary Table 1. The average lifetime (*τ*_*aver*_) is calculated according to the Eqs (equations)^52–54^, where *τ*_*1*_ refers to the fitted short lifetime, *τ*_*2*_ refers to the fitted long lifetime. *τ*_*1*_ and *τ*_*2*_ may originate from parity-allowable transitions and parity-forbidden transitions respectively^8,9,55^. *A*_*1*_ and *A*_*2*_ are the weights of two exponential functions, *Φ* refers to *PLQY*, *K*_*R*,_ and *K*_*NR*_ refers to the radiation transition rate and the non-radiative transition rate, respectively. It reveals that the sample of x = 0 has the biggest value of *τ*_*ave*_, the sample of x = 0.4 has the biggest value of *K*_*R*_, and the sample of x = 0.6 has the biggest value of PLQY. when x = 0.6, the maximum value of radiative recombination proportion (*K*_*R*_*/K*_*NR*_) is 0.274. Combined Eq. (3) in the Supplementary information, the larger the *K*_*R*_*/K*_*NR*_ value, the larger the *Φ* value. *Φ* is proportional to *K*_*R*_*/K*_*NR*_. The *K*_*R*_*/K*_*NR*_ value can demonstrate the relationship between radiative recombination and non-radiative recombination. For example, the sample of x = 0.4, and x = 0.6, the obtained *K*_*R*_*/K*_*NR*_ value are 0.272, and 0.274 respectively. Their PLQY are 21.26%, and 21.6% respectively, which is the relatively high PLQY value among the six samples. The enhanced PLQY, nearly twice as high as other samples, demonstrates the increase of Na^+^ incorporation content can raise the radiative recombination proportion. Overall, *K*_*R*_*/K*_*NR*_ value increases from 0.116 to 0.274, and then reduces to 0.122. The change values of *Φ* present a trend of first rising and then falling. It increases from 10.39 to 21.6%, which suggests that Na^+^ doping plays a significant role in enhancing the radiative recombination probability, thus the PLQY is improved, and it then reduces to 10.67%. From this, it can be seen that the *Φ* value is influenced by the *K*_*R*_*/K*_*NR*_ value. The sample of x = 0.6 possesses the highest *K*_*R*_*/K*_*NR*_ value. Combined Fig. 1c, TRPL result and PL spectrum of Cs_2_Ag_0.4_Na_0.6_In_0.5_Bi_0.5_Cl_6_ both prove that x = 0.6 is the suitable Na^+^ doping ratio.

**Figure 2:**
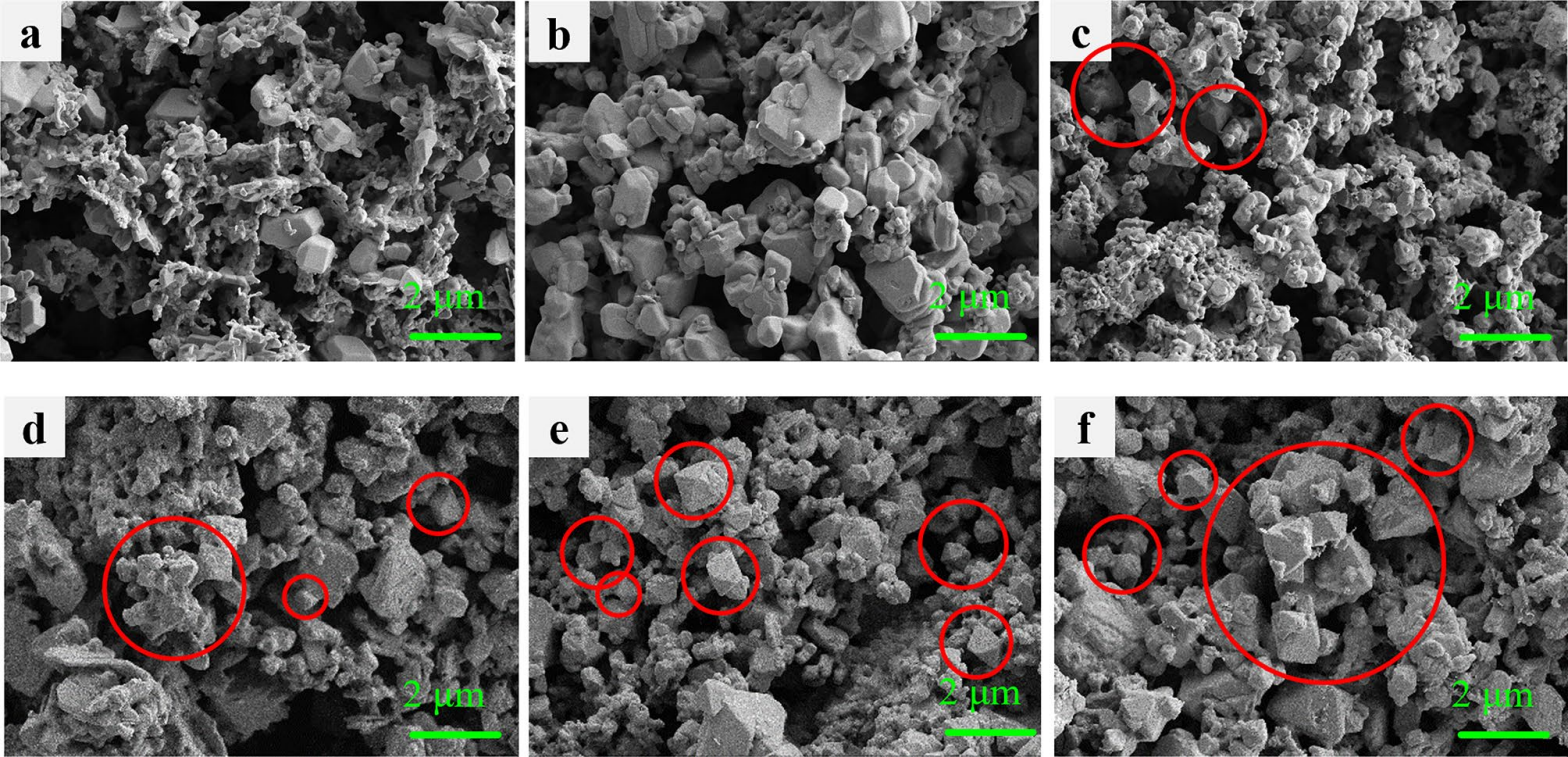
SEM images. (**a**), Cs_2_AgIn_0.5_Bi_0.5_Cl_6_. (**b**) Cs_2_Ag_0.8_Na_0.2_In_0.5_Bi_0.5_Cl_6_. (**c**) Cs_2_Ag_0.6_Na_0.4_In_0.5_Bi_0.5_Cl_6_. (**d**) Cs_2_Ag_0.4_Na_0.6_In_0.5_Bi_0.5_Cl_6_. (**e**) Cs_2_Ag_0.2_Na_0.8_In_0.5_Bi_0.5_Cl_6_. (**f**), Cs_2_NaIn_0.5_Bi_0.5_Cl_6_.

Supplementary Fig. 5is the SEM image of Cs_2_AgInCl_6_. Cs_2_AgInCl_6_ tends to grow into homogeneous films with a large number of holes and cracks. In Fig. 2, SEM was used to analyze the morphology evolution of the films at different Na content. Interestingly, it can be seen that the morphology of the films changes from irregular shapes, such as flaky, and strip-shaped to octahedral shapes. The irregular shapes, such as the bulk-shaped or particle-shaped products may achieve through the aggregation of the nanocrystals. This is due to the effect of soft agglomeration of electrostatic attraction or van der Waals forces. Compared with Supplementary Fig. 5, the SEM images of Fig. 2a–f doped with Na and Bi show that it is composed of separated particles with uncertain appearance and size. Starting with the sample with x = 0.4, some obvious octahedral shapes began to appear as circled in the picture. As the value of x increases, the SEM image shows more and more octahedral. Octahedral shaped crystals are usually considered to be more complete and regular crystal forms, and their crystal quality may be higher. They may have lower surface energy and fewer defects compared to other shapes, which may have a positive impact on their optoelectronic performance^30^. However, even though the x = 1.0 film has the most octahedra in the SEM, its luminescence efficiency didn’t improve because it does not contain Ag and cannot be alloyed with Na for luminescence. Combined the Fig. 1a, it can be seen that the (220) peak intensity of the sample with x = 0.6 is the highest. It may indicate that the crystal structure of the x = 0.6 sample has a better crystallinity with a more ordered arrangement of atoms in the (220) crystal plane. Next, the energy dispersive X-ray spectroscopy (EDS) was carried out to detect the element distribution of the Cs_2_Ag_1 − x_Na_x_In_0.5_Bi_0.5_Cl_6_ films by verifying Cs, Ag, Na, In, Bi, and Cl elements. The corresponding EDS spectra are displayed in Supplementary Fig. 6. According to the element ratios measured by EDS (Supplementary Table 2) and Supplementary Fig. 8, some potential patterns can be discovered. Intriguingly, before adding Na^+^, the content of Ag is very low, making it difficult to enter the system. After the addition of Na, it greatly promotes the integration of Ag into the system. With Na incorporation increases, the incorporation amount of Ag tends to promote, as well as Bi, which is favorable for Bi^3+^ successfully substituting part of In^3+^. It should be noted that this phenomenon is based on appropriate Na incorporation. For example, when x increases from 0 to 0.2, the percentage of Ag increases from 0.71 to 1.94%, Bi^3+^ increases from 3.02 to 6.1%. According to the observations of atomic content and element ratio in Supplementary Fig. 7a and b, we made a conjecture that a certain amount of Na doping into the DP structure can promote Ag and Bi content in the system.

**Figure 3:**
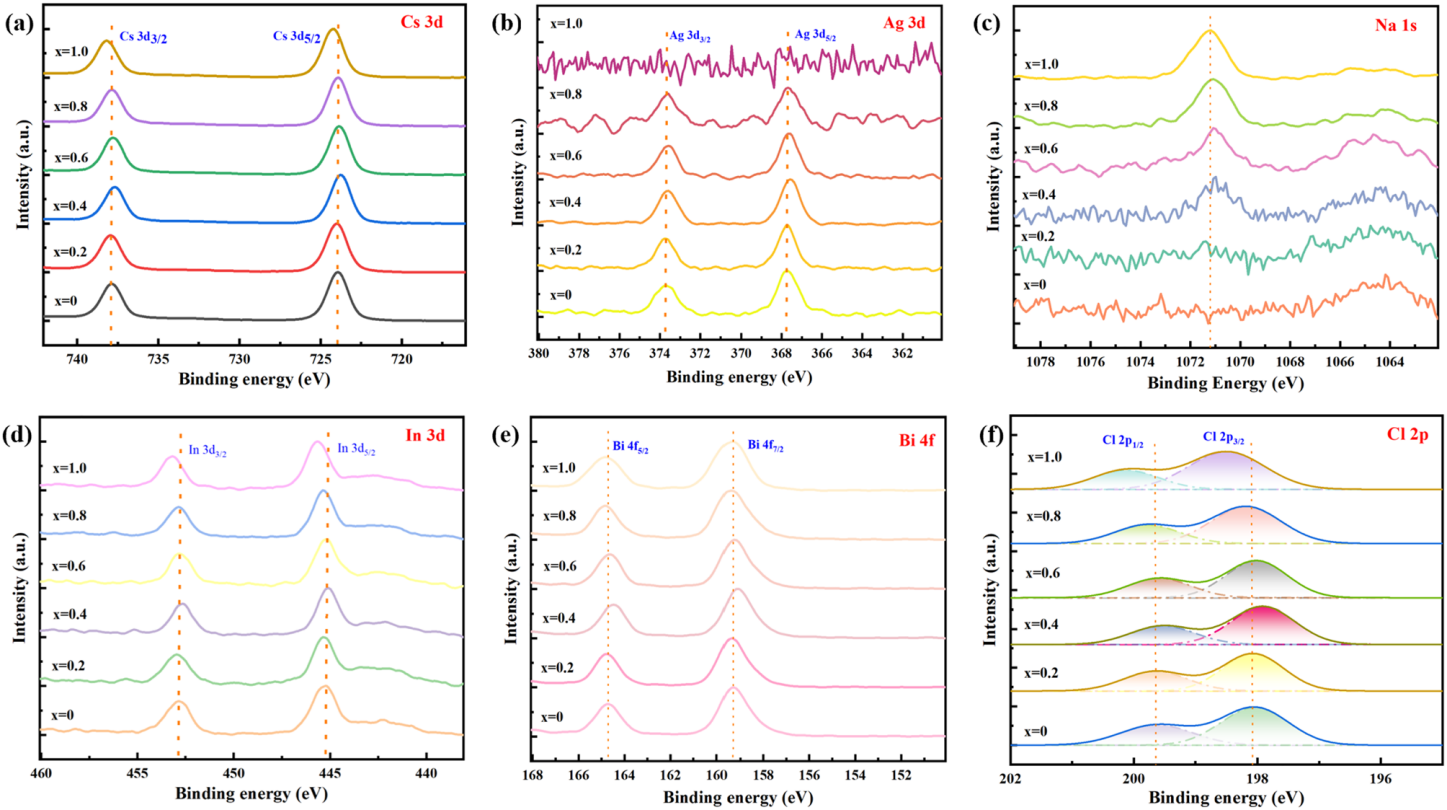
Results of the XPS analyses conducted on Cs_2_Ag_1 − x_Na_x_In_0.5_Bi_0.5_Cl_6_ films. (a) Cs 3*d*. (**b**), Ag 3*d*. (**c**) Na 1* s*. (**d**) In 3*d*. (**e**), Bi 4*f*. (**f**) Cl 2*p*.

Figure 3a–f show the XPS results of the composition of the Cs_2_Ag_1 − x_Na_x_In_0.5_Bi_0.5_Cl_6_ films. Cs, Ag, In, Bi, Cl all had two split peaks locating at around 737.88 eV and 723.88 eV, 373.68 eV and 367.78 eV, 452.88 eV and 445.18 eV, 164.68 eV and 159.28 eV, 199.63 eV and 198.12 eV, which were assigned to Cs 3*d*_3/2_ and Cs 3*d*_5/2_, Ag 3*d*_3/2_ and Ag 3*d*_5/2_, In 3*d*_3/2_ and In 3*d*_5/2_, Bi 4*f*_5/2_ and Bi 4*f*_7/2_, Cl 2*p*_1/2_, Cl 2*p*_3/2_ electronic levels, respectively. Regarding to Cs, the change in peak position is minimal as shown in Fig. 3a. This indicates that Cs are relatively stable in the Cs_2_Ag_1 − x_Na_x_In_0.5_Bi_0.5_Cl_6_ system regardless of Na^+^ doping. Figure 3c depicts a peak locates at 1071.2 eV corresponding to Na 1 s. Starting from x = 0.4, the peak of Na 1* s* is more pronounced, which suggests some Ag^+^ ions were successfully substituted by Na^+^ ions as expected. Similarly, Fig. 3b also confirms this conclusion. When x = 1.0, there is no Ag element in the Cs_2_NaIn_0.5_Bi_0.5_Cl_6_ film. So, it’s obviously there aren’t Ag 3*d*_3/2_ and Ag 3*d*_5/2_ peaks. Interestingly, it was found that In 3*d*_3/2_ and In 3*d*_5/2_ peaks of Cs_2_NaIn_0.5_Bi_0.5_Cl_6_ shift towards high binding energy direction compared to other films (shown in Fig. 3d). This may be because more [InCl_6_] octahedra were formed in the pure Na system. Additionally, Fig. 3f shows Cl 2*p*_1/2_ and Cl 2*p*_3/2_ move towards the direction of high binding energy as Na^+^ doped. The reason for this may be due to excessive Na doping, resulting in an increase in [NaCl_6_] octahedra.

### Bi^3+^ doping

**Figure 4:**
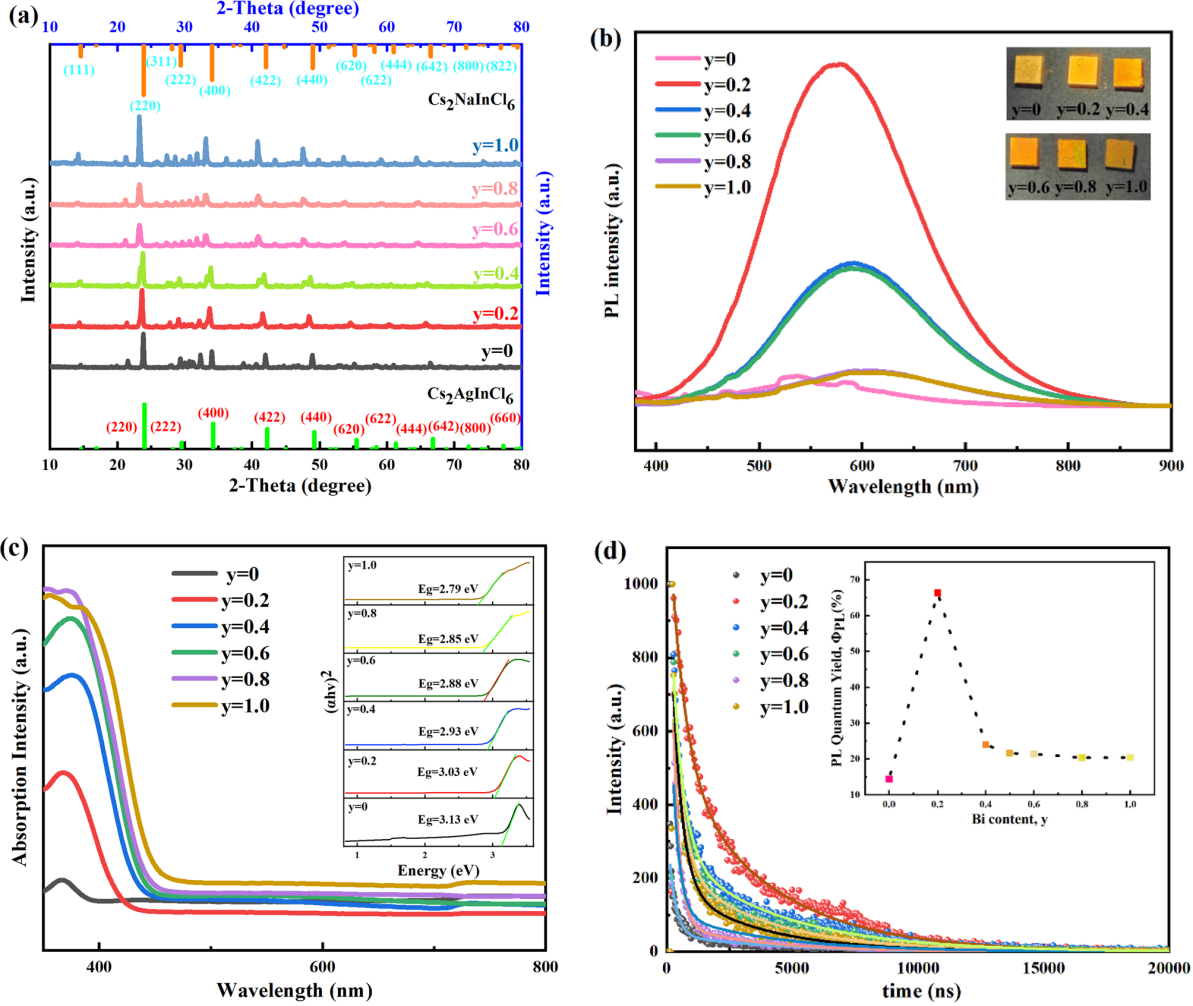
Structural characterizations and optical property of Cs_2_Ag_0.4_Na_0.6_In_1 − y_Bi_y_Cl_6_ films. (**a**) XRD patterns of Cs_2_Ag_0.4_Na_0.6_In_1 − y_Bi_y_Cl_6_ at different value of y. (**b**) PL spectra of films at increasing y from y = 0 to y = 1 with 0.2 steps. The inset picture are the samples of y = 0.2, 0.4, 0.6, 0.8, 1.0. They were excited under 350 nm ultraviolet. (**c**) Optical absorption spectra of Cs_2_Ag_0.4_Na_0.6_In_1 − y_Bi_y_Cl_6_ films at increasing y from y = 0 to y = 1 with 0.2 steps, and Tauc plots (the inset picture). (**d**) Time-resolved PL decay curves of Cs_2_Ag_0.4_Na_0.6_In_1 − y_Bi_y_Cl_6_ measured at 590 nm, λ_exc_ = 350 nm, except for y = 0 sample (λ_exc_ = 310 nm, measured at 540 nm), and PLQY results (the inset picture).

Based on PL and PLQY of Cs_2_Ag_1 − x_Na_x_In_0.5_Bi_0.5_Cl_6_ films, we set x = 0.6 and change the value of y in the system of Cs_2_Ag_0.4_Na_0.6_In_1 − y_Bi_y_Cl_6_ (y = 0, 0.2, 0.4, 0.6, 0.8, 1.0). The composition of the Cs_2_Ag_0.4_Na_0.6_In_1-y_Bi_y_Cl_6_ films was modulated by systematically changing the value of y from 0 to 1. The XRD patterns of Cs_2_Ag_0.4_Na_0.6_In_1 − y_Bi_y_Cl_6_ films are shown in Fig. 4a. It demonstrates that these films are still in cubic Fm-3 m DP structure after adding Bi^3+^ dopant. We can notice that the (220) diffraction peak gradually offset towards the direction where the value of 2θ decreases, from 23.87 to 23.27°. According to the Bragg equation, when some Bi^3+^ ions (1.17 Å) replace the position of In^3+^ ions (0.94 Å), the crystal cell parameters will increase, and the *d* value will also increase. Therefore, the value of θ decreases. The XRD diffraction peak will shift to the left. Figure 4b shows the PL spectra of Cs_2_Ag_0.4_Na_0.6_In_1 − y_Bi_y_Cl_6_ films. The PL intensity of the y = 0.2 sample is much greater than other samples. The inset pictures show the luminescence of Cs_2_Ag_1 − x_Na_x_In_1 − y_Bi_y_Cl_6_ films under excitation of a 365 nm ultraviolet lamp. Figure 4c is the absorption spectra of Cs_2_Ag_0.4_Na_0.6_In_1 − y_Bi_y_Cl_6_ films. Tauc plots demonstrate that the bandgap value continues to decrease as the y value increases. It is shown in the inset image of Fig. 4c. This is in stark contrast with the bandgap phenomenon of Cs_2_Ag_1 − x_Na_x_In_0.5_Bi_0.5_Cl_6_ samples. These bandgap results maintain a range from 2.79 to 3.13 eV, which is in good agreement with previous studies ^25,37,43^.

From Fig. 4d, when y = 0.2, Cs_2_Ag_0.4_Na_0.6_In_0.8_Bi_0.2_Cl_6_ film shows the highest value of PLQY (66.38%). Similarly, time-resolved PL measurements of Cs_2_Ag_0.4_Na_0.6_In_1 − y_Bi_y_Cl_6_ films were also carried out. The time-resolved PL spectra are shown in Fig. 4d. In Supplementary Table 3, *K*_*R*_/*K*_*NR*_ increase from y = 0 to y = 0.2. It can be seen that *K*_*R*_*/K*_*NR*_ up to19.8 at y = 0.2, which is the highest data among all of the six Cs_2_Ag_1-x_Na_x_In_0.5_Bi_0.5_Cl_6_ samples. Its PLQY, 66.38%, is also the highest value in all samples. The extremely high *K*_*R*_*/K*_*NR*_ and PLQY manifest that radiation recombination occupies a more important position than non-radiation recombination in Cs_2_Ag_0.4_Na_0.6_In_0.8_Bi_0.2_Cl_6_ sample. When y = 0.4, the value of* K*_*R*_/*K*_*NR*_ suddenly drops to 0.313, then it decreases from y = 0.6 to y = 1.0, from 0.313 to 0.258. Combined Fig. 4b,d, PL and PLQY are associated with non-radiative loss process. These curves also have a trend of first rising and then falling. There lies an interesting phenomenon that the effect of Bi^3+^ cations doping on PL and PLQY is more significant than Na^+^ ion doping. Compared Fig. 1f with Fig. 4d, for Na^+^ doping, the PLQY value varies within the range of 10.39–21.6%. While for Bi^3+^ doping, PLQY value changes more dramatically. It rapidly increased from 14.37 to 66.38%, and then began to steadily decline, maintaining a value above 20%. Firstly, this is attribute to the appropriate Na^+^ and Bi^3+^ doping ratios found in the Cs_2_Ag_1−__x_Na_x_In_1 − y_Bi_y_Cl_6_ system. Secondly, it is due to the further promotion of the radiative recombination process upon Bi^3+^ doping. y = 0.2, y = 0.4, y = 0.6, y = 0.8, and y = 1.0 samples all exhibit higher *K*_*R*_ and *K*_*R*_*/K*_*NR*_ than the sample of y = 0. Considering their same Na^+^ doping ratio, an obvious difference between y = 0 and other samples is that it does not contain Bi^3+^ content. For this reason, it is speculated that radiative recombination ratio can be promoted with Bi^3+^ doping. Thridly, the Bi^3+^ incorporation is considered to improve crystal quality and promote exciton localization^29^. The *K*_*NR*_ value of the sample with y = 0 is more than twice that of the sample with y = 0.2. With Bi^3+^ doping, the radiative localization is promoted, defects of the films are passivated and within a certain range, non-radiative recombination loss is suppressed, which further enhances the PLQY^29^.

The PLQY value of the sample with y = 0.2 is more than three times that of other samples. PLQY has a huge decrease from y = 0.4. Two factors may account for the decreased PLQY upon further increasing the Bi content. *K*_*R*_ value began to sharply decline from the sample y = 0.4. According to Eq. (1), Fermi’s golden rule, *K*_*R*_ is proportional to the square of the transition dipole moment.The decrease in transition dipole moment caused by the orbital spatial overlap between electrons and holes for STEs may be the first reason, which affects electron–hole recombination and reduces the probability of electronic transitions^29^. The second reason is the increased non-radiative loss, which can be seen from the gradually increasing *K*_*NR*_ value. It may be attributed to the phonon emission from the recombination between some photoexcited electrons and holes. In summary, all data prove that selecting x = 0.6, and y = 0.2 as the Na optimal alloying and Bi doping parameters is suitable for the purpose of high-quality and high-performance thin films.

1 $$K_{R} = \frac{{4\pi^{3} }}{{3\varepsilon_{0} hc^{3} }} \cdot \frac{{\left| {\mu_{fi} } \right|^{3} }}{{\lambda^{3} }}$$

**Figure 5:**
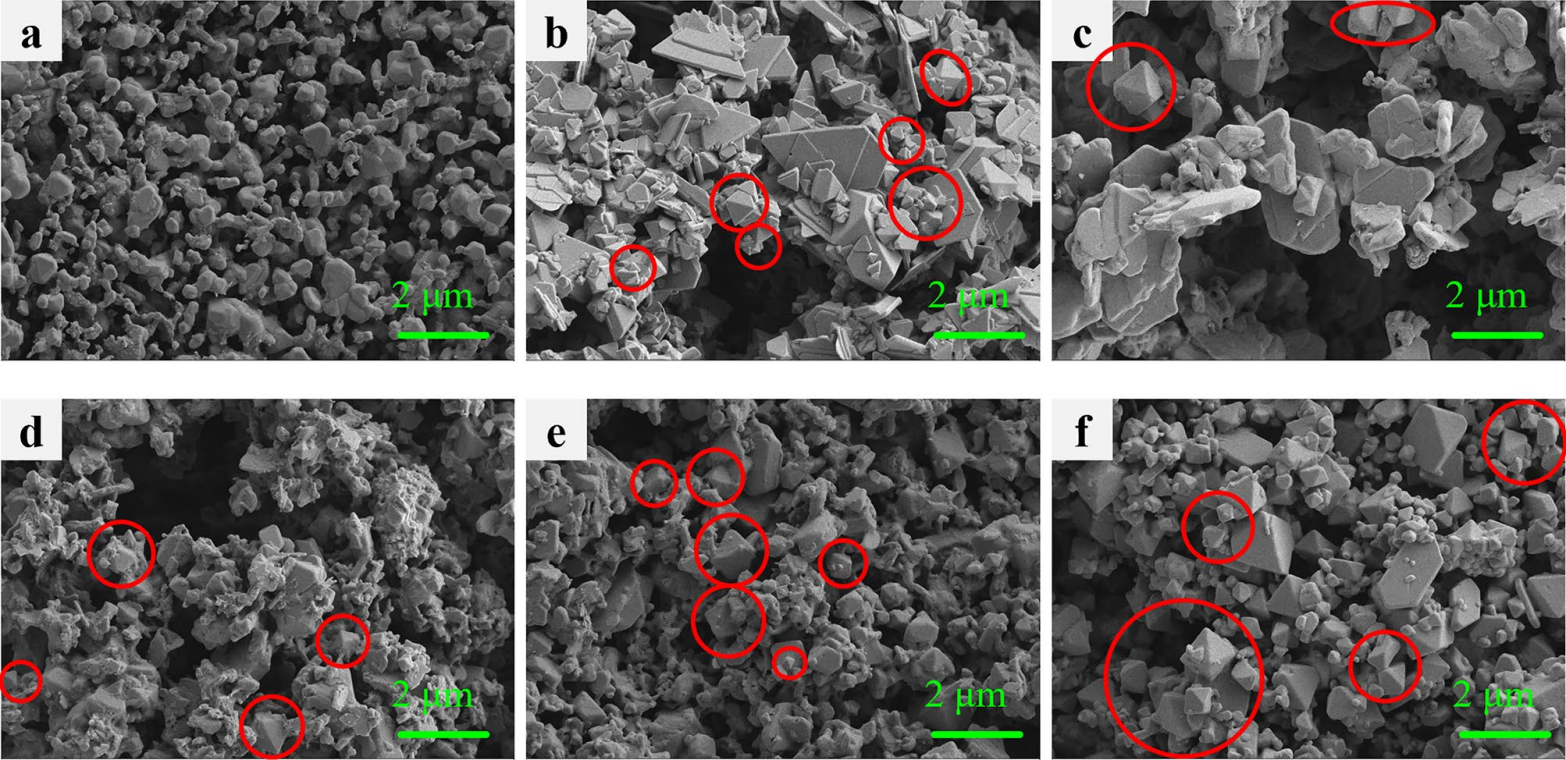
SEM images. (**a**) Cs_2_Ag_0.4_Na_0.6_InCl_6_. (**b**) Cs_2_Ag_0.4_Na_0.6_In_0.8_Bi_0.2_Cl_6_. (**c**) Cs_2_Ag_0.4_Na_0.6_In_0.6_Bi_0.4_Cl_6_. (**d**) Cs_2_Ag_0.4_Na_0.6_In_0.4_Bi_0.6_Cl_6_. (**e**), Cs_2_Ag_0.4_Na_0.6_In_0.2_Bi_0.8_Cl_6_. (**f**) Cs_2_Ag_0.4_Na_0.6_BiCl_6_.

Figure 5a–f show the SEM images of Cs_2_Ag_0.4_Na_0.6_In_1 − y_Bi_y_Cl_6_ films. Figure 5a shows that the Cs_2_Ag_0.4_Na_0.6_InCl_6_ film consists of irregular nanoparticles. It could be noticed that the sample of y = 0.2 began to have octahedral shapes, which indicates the doping of Bi^3+^ can change the morphology of the films, appearing as octahedral^30^. Generally, most of these films are mixed in flakes, rods, and octahedra. As the y value increases, the grains gradually grow from flakes to octahedra. The increase in Bi^3+^ cations doping is beneficial for the growth of more octahedra in the Cs_2_Ag_0.4_Na_0.6_In_1 − y_Bi_y_Cl_6_ system. In Fig. 4b, the PL intensity of the y = 0.2 sample is the highest, and there is not much difference in PL intensity between the y = 0.4 sample and y = 0.6 sample, y = 0.8 sample and y = 1.0 sample. In Fig. 5e,f, It can be observed that the crystals has grown. The decrease in PL strength may be due to the introduction of lattice defects or deformation caused by grain growth. In order to understand the effect of Na^+^ and Bi^3+^ doping on lattice parameters, the crystal data of (220) crystal plane were analyzed based on the XRD patterns in Figs. 1a and 4a, which is shown in Supplementary Tables 4 and 5. Futhermore, the Williamson–Hall analysis based on XRD patterns (as shown in Supplementary Tables 6 and 7) suggested the average grain size was about 64.57 nm for y = 0.2 sample. The data in Supplementary Tables 6 and 7 are obtained based on the fitting results in Supplementary Figs. 8 and  9, respectively. EDS spectra and the element proportions of Cs_2_Ag_0.4_Na_0.6_In_1 − y_Bi_y_Cl_6_ films are shown in Supplementary Fig. 10 and Supplementary Table 8. According to Supplementary Fig. 11 and Supplementary Table 8, with y increase from 0 to 1, the doping percentage of Bi^3+^ continues to increase, from 0 to 10.33%. In general, the ratio of Ag also increases as the incorporation amount of Bi^3+^. This indicates that to a certain extent, an increase in Bi^3+^ content will also promote the content of Ag in the system. For Cs_2_Ag_0.4_Na_0.6_In_0.8_Bi_0.2_Cl_6_ film, according to Supplementary Fig. 11a, the atomic percentage of Cs, Ag, Na, In, Bi, Cl are 24.15%, 1.81%, 7.2%, 9.94%, 1.57%, 55.33%, respectively, which means the actual proportion of elements of this sample is Cs_2_Ag_0.15_Na_0.60_In_0.82_Bi_0.13_Cl_4.58_.

**Figure 6:**
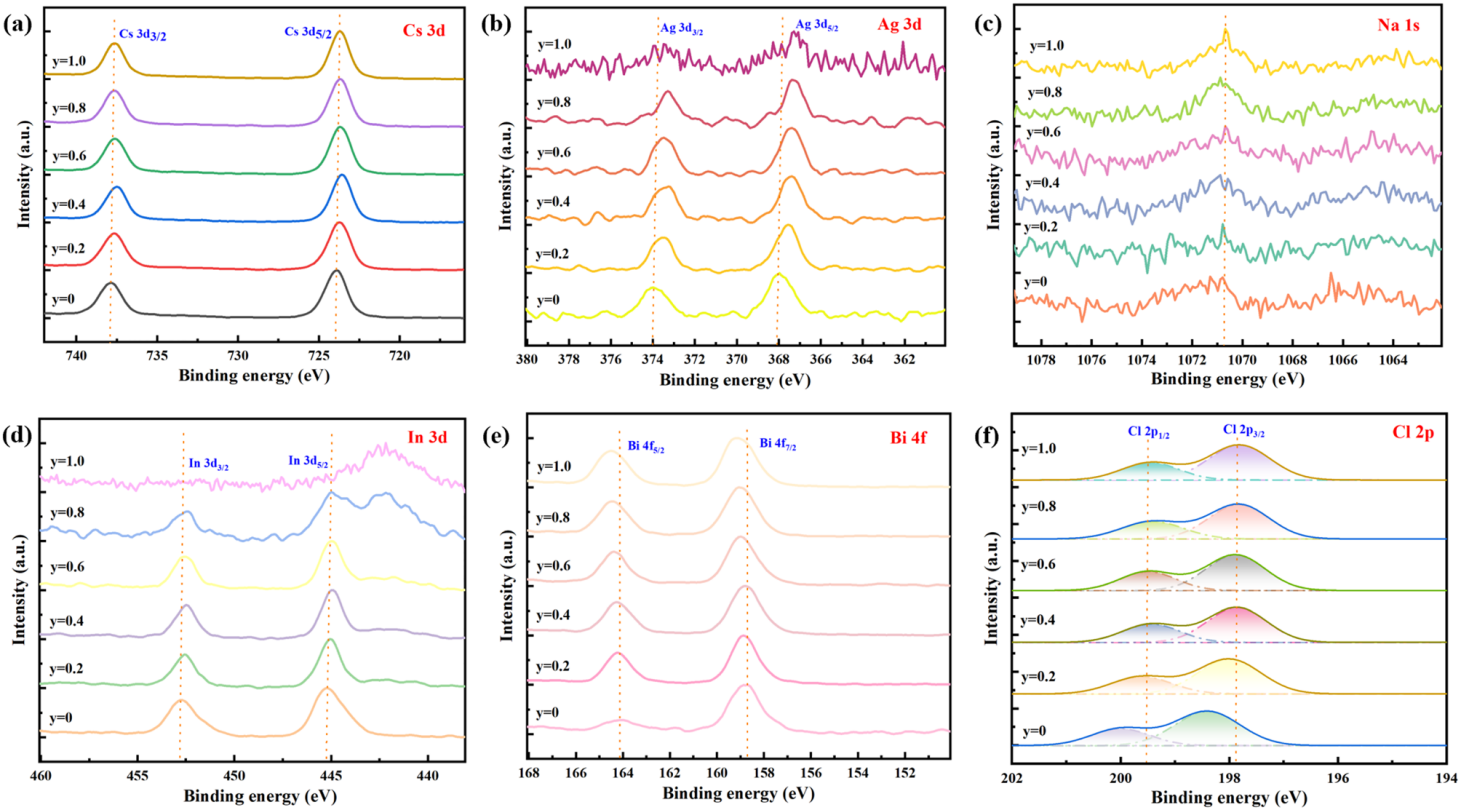
Results of the XPS analyses conducted on Cs_2_Ag_0.4_Na_0.6_In_1 − y_Bi_y_Cl_6_ films. (**a**) Cs 3*d*. (**b**), Ag 3*d*. (**c**), Na 1* s*. (**d**), In 3*d*. (**e**) Bi 4*f*. (**f**) Cl 2*p*.

For Cs_2_Ag_0.4_Na_0.6_In_1-y_Bi_y_Cl_6_ films, Fig. 6a to f show that Cs, Ag, In, Bi, Cl all had two split peaks locating at around 737.88 eV and 723.88 eV, 373.98 eV and 367.98 eV, 452.88 eV and 445.18 eV, 164.18 eV and 158.68 eV, 199.51 eV and 197.87 eV, which were assigned to Cs 3*d*_3/2_ and Cs 3*d*_5/2_, Ag 3*d*_3/2_ and Ag 3*d*_5/2_, In 3*d*_3/2_ and In 3*d*_5/2_, Bi 4*f*_5/2_ and Bi 4*f*_7/2_, Cl 2*p*_1/2_ and Cl 2*p*_3/2_ electronic levels, respectively. The peak positions of Cs 3*d*_3/2_ and Cs 3*d*_5/2_ have hardly change, which proves that Cs^+^ is relatively stable in the Cs_2_Ag_0.4_Na_0.6_In_1-y_Bi_y_Cl_6_ system. Notably, Ag 3*d*_3/2_ and Ag 3*d*_5/2_ move towards the direction of low binding energy from y = 0 to y = 1.0. When Bi^3+^ partially substituted In^3+^, doping into the lattice, it may squeeze the [AgCl_6_] octahedrons. So, for Ag^+^, they display a trend moving towards the low binding energy. Na 1* s* peak locate at around 1070.78 eV. For In^3+^, there is an evident change at y = 0.8 and y = 1.0. This indicates that more Bi^3+^ cations have replaced In^3+^ cations, thus reducing the binding energy of In 3*d*_3/2_ and In3*d*_5/2_. Instead, Bi 4*f*_5/2_, Bi 4*f*_7/2_ move toward the direction of high binding energy from y = 0 to y = 1.0 as shown in Fig. 6e. For Cl 2*p*_1/2_ and Cl 2*p*_3/2_, the Bi^3+^ doping samples had a lower binding energy than that of non-doping sample as observed in Fig. 6f, which may derive from the environment change after Bi^3+^ doping.

### Stability and the light emitting devices

**Figure 7:**
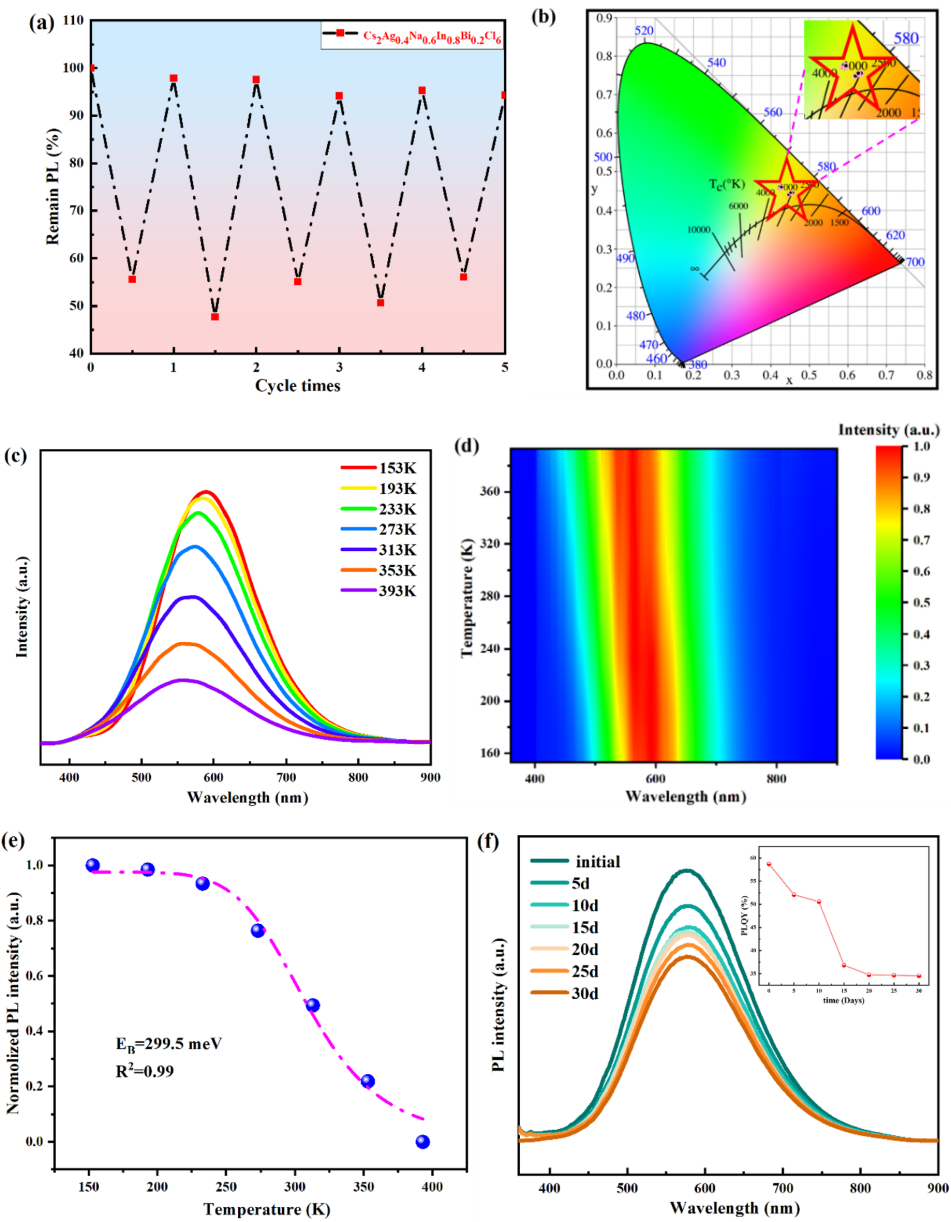
Stability of Cs_2_Ag_0.4_Na_0.6_In_0.8_Bi_0.2_Cl_6_ film. (**a**) Heating/cooling PL tests of the Cs_2_Ag_0.4_Na_0.6_In_0.8_Bi_0.2_Cl_6_ sample at a temperature of 20–100 °C. (**b**) CIE chart of the Cs_2_Ag_0.4_Na_0.6_In_0.8_Bi_0.2_Cl_6_ sample during the thermal stability test. (**c**) Photoluminescence spectra of Cs_2_Ag_0.4_Na_0.6_In_0.8_Bi_0.2_Cl_6_ film measured at different temperatures from 153 to 393 K. (**d**) Contour plot. e, Integrated PL emission intensity of Cs_2_Ag_0.4_Na_0.6_In_0.8_Bi_0.2_Cl_6_ film as a function of temperature from 153 to 393 K. (**f**) Photoluminescence spectra of Cs_2_Ag_0.4_Na_0.6_In_0.8_Bi_0.2_Cl_6_ film measured at fixed interval (5 days) in 30 days, the inset picture is the PLQY of the sample.

Then we conducted a cold and hot cycling experiment to test the thermal stability of the Cs_2_Ag_0.4_Na_0.6_In_0.8_Bi_0.2_Cl_6_ sample. One cycle refers to the temperature heated from 20 to 100 °C and then cooled from 100 to 20 °C. The Cs_2_Ag_0.4_Na_0.6_In_0.8_Bi_0.2_Cl_6_ sample was measured at five cycles and the remaining PL intensity of the sample at each cycle point was recorded as shown in Fig. 7a. It can be easily seen that the remaining PL emission intensity of the Cs_2_Ag_0.4_Na_0.6_In_0.8_Bi_0.2_Cl_6_ sample decreased as the temperature increased during the heating process and increased during the cooling process. During five cycles, the remaining PL intensity decreased slightly, but the overall change was not significant. When five thermal cycles are finished, the sample still preserve their relative emission intensity of 94.3%. Figure 7b displays a Commission International de I’Eclairage color coordinates (CIE) chart of the Cs_2_Ag_0.4_Na_0.6_In_0.8_Bi_0.2_Cl_6_ sample during the thermal stability test. From the picture, we can see that the color coordinates are concentrated in the warm-yellow light region, implying that the thermal cycling process didn’t have a significant impact on the luminescent color of the sample. From the correlated color temperature (CCT) chart (Supplementary Fig. 12), it also declares that the sample emitted relatively stable light. Moreover, we conducted thermogravimetric analysis (TGA) and differential scanning calorimeter (DSC) analysis of the Cs_2_Ag_0.4_Na_0.6_In_0.8_Bi_0.2_Cl_6_ slurry to further measure its thermal stability (Supplementary Fig. 13). The DSC curve indicates our sample began to crystallize around 170 °C and began to decompose around 560 °C, which is consistent with the pattern in previous literature^29,31^. Such high thermal stability gets benefit from the resistance of thermal stress for all-inorganic perovskites compared to inorganic–organic hybrid perovskites. The TGA curve displays a big mass drop from 22 to 178 °C, which may drive from the evaporation of DMSO in the slurry. Figure 7c displays the normalized PL spectra of the Cs_2_Ag_0.4_Na_0.6_In_0.8_Bi_0.2_Cl_6_ sample measured at the temperature range from 153 to 393 K. The contour plot is also shown in Fig. 7d. Interestingly, PL spectra occurs a blueshift phonomenon, which should attribute to the electron–phonon interraction and the crystal lattice thermal expansion^56,57^. Due to the thermally activated nonradiatie recombination, the PL intensity gradually decreased with the increasement of the temperature.

To clarify the internal mechanism of the effect of the temperature on the luminescence intensity of the film, the Arrhenius Eq. (2) was used to fit the curve that the normolized PL intensity as a function of temperature for Cs_2_Ag_0.4_Na_0.6_In_0.8_Bi_0.2_Cl_6_ sample, which is shown in Fig. 7e.

2 $$I(T) = \frac{{I_{0} }}{{1 + Ae^{{ - E_{b} /K_{b} T}} }}$$

where *I(T)* and *I*_*0*_ refer to the PL intensities at temperatures TK and 0 K, respectively. *A* is the fitting constant, *E*_*b*_ is the exciton binding energy, and *K*_*b*_ is the Boltzmann constant. The fitting results reveals the *E*_*b*_ value is 299.5 meV. With a certain range, the large the value, the more favorable it is for excition survival at RT and even a high temeprtaure^58–60^. It is noticed that the PL spectral bandwidth widens with the rising of temperature, which is related to electron–phonon coupling. More specifically, acoustic phonons and longitudinal optical (LO) phonons can affect PL bandwidth. In the high-temperature region, PL broadening is mainly controlled by LO phonons. As the temperature further decreases, there is a crossover between acoustic phonons and LO phonons, thus the interaction with acoustic phonons controls the broadening^61^. Figure 7f depicts the PL spectra, PLQY of Cs_2_Ag_0.4_Na_0.6_In_0.8_Bi_0.2_Cl_6_ sample under the continuously 365 nm UV irradiation in 30 days to evaluate its photostability. After a continuous irradiation of 720 h, 31.9% of the initial PL intensity, 41.2% of the initial PLQY were diminished.

**Figure 8:**
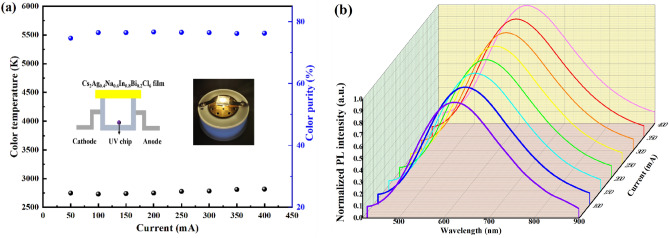
Stability of Cs_2_Ag_0.4_Na_0.6_In_0.8_Bi_0.2_Cl_6_ LED. (**a**) CCT, color purity of the Cs_2_Ag_0.4_Na_0.6_In_0.8_Bi_0.2_Cl_6_ LED with different driving currents (from 50 to 400 mA) under 365 nm UV irradiation. The insets show the structure diagram of the LED and a photo of the Cs_2_Ag_0.4_Na_0.6_In_0.8_Bi_0.2_Cl_6_ LED. (**b**) Normalized PL spectra of the Cs_2_Ag_0.4_Na_0.6_In_0.8_Bi_0.2_Cl_6_ LED.

In order to explore the possibility of applying our materials to LED field, we put Cs_2_Ag_1 − x_Na_x_In_1 − y_Bi_y_Cl_6_ samples on ultraviolet light chips (365 nm) to fabricate their corresponding LEDs. Supplementary Fig. 14a, b record the Cs_2_Ag_1 − x_Na_x_In_0.5_Bi_0.5_Cl_6_ samples and Cs_2_Ag_0.4_Na_0.6_In_1 − y_Bi_y_Cl_6_ samples CIE chart respectively. Supplementary Fig. 15a,b record their CCT respectively. Supplementary Table 9 presents the CCT and CRI of the Cs_2_Ag_0.4_Na_0.6_In_1-y_Bi_y_Cl_6_ LEDs. It is noticed that the CCT of Cs_2_Ag_1 − x_Na_x_In_0.5_Bi_0.5_Cl_6_ samples locate in a range from 2969 to 5913 K, the CCT of Cs_2_Ag_0.4_Na_0.6_In_1-y_Bi_y_Cl_6_ samples locate in a range from 2727 to 3517 K, which manifests the samples prepared through our fabrication approach could adjust the color of LED light within a wide range. Importantly, it will be highly potential for the application in making LEDs. Figure 8a,b show the CCT, color purity, and PL spectra of Cs_2_Ag_0.4_Na_0.6_In_0.8_Bi_0.2_Cl_6_ LED under different driving currents, respectively. For example, under 100 mA, the CIE color coordinate is (0.48, 0.44), CCT is 2732 K, the high color rendering index (CRI) is 85.5. With the increase of the driving current, the CCT and the color purity nearly had no change. However, the increase of the PL intensity was clearly observed. Additionally, CIE color coordinates of the Cs_2_Ag_0.4_Na_0.6_In_0.8_Bi_0.2_Cl_6_ LED is shown in Supplementary Fig. 16.

## Conclusions

In conclusion, we have demonstrated a facile blade coating avenue, with alkali metal Na^+^ and Bi^3+^ doping into Cs_2_AgInCl_6_, via parament design optimization to prepare Cs_2_Ag_1 − x_Na_x_In_1 − y_Bi_y_Cl_6_ films. By Na^+^ alloying (60% mol) and Bi^3+^ alloying (20% mol), Cs_2_Ag_0.4_Na_0.6_In_0.8_Bi_0.2_Cl_6_ film exhibited a broad spectrum warm-yellow PL with a quantum yield of 66.38%. It is found that doping with Na^+^ and Bi^3+^ can break parity-forbidden transition, decrease the defect density and promote the radiative recombination, and greatly improve the luminescence performance of the samples. To verify its photostability and thermal stability, the corresponding experiments were conducted. After 720 h continuous ultraviolet irradiation, only 31.9% emission decay. And after five thermal cycles, the PL intensity is only reduced to 5.7% of the initial value. Furthermore, Cs_2_Ag_0.4_Na_0.6_In_0.8_Bi_0.2_Cl_6_ film was used to fabricate a LED, which shows the CIE color coordinates of (0.48, 0.44), the CCT of 2732 K, and the CRI of 85.5 under 100 mA, falling in the warm-yellow light region. More work and research should focus on approaches to improve the pinhole and defect of the Cs_2_Ag_0.4_Na_0.6_In_0.8_Bi_0.2_Cl_6_ film. In addition, due to the broadband warm-yellow emission, it will be involved to the full-spectrum lighting devices in our future work.

## Methods

### Materials

CsCl (99.9%, Adamas-beta), AgCl (98%, Adamas-beta), NaCl (analytical pure, Wuxi Yatai United Chemical Co., Ltd), BiCl_3_ (analytical pure, Aladdin), InCl_3_ (99.99%, Adamas-beta), dimethyl sulfoxide (DMSO, > 99%, Sinopharm Chemical Reagent Co., Ltd, China). All chemicals were used as received without further purification.

### Synthesis of Cs_2_Ag_1 − x_Na_x_In_1 − y_Bi_y_Cl_6_ films

**Figure 9:**
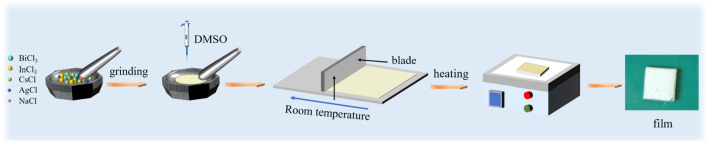
Experiment synthesis procedures of Cs_2_Ag_1 − x_Na_x_In_1 − y_Bi_y_Cl_6_ films.

As illustrated in Fig. 9, firstly, CsCl, AgCl and NaCl, InCl_3_ and BiCl_3_ molar ratio 2:1:1 was put into a mortar. Then fully ground the mixture, adding DMSO solvent to form a slightly sticky paste. Through fully ground the mixture, a facile and low-cost blade coating method was used to transfer the as-prepared slurry to glass substrates and subsequently heated at 150 °C for 10 min on a hot plate for the evaporation of DMSO and crystallization for Cs_2_Ag_1 − x_Na_x_In_1 − y_Bi_y_Cl_6_ (x = 0, 0.2, 0.4, 0.6, 0.8 and 1.0), (y = 0, y = 0.2, y = 0.4, y = 0.6, y = 0.8, y = 1.0) films. For example, when x = 0.2, y = 0.5, typically, 4 mmol CsCl (0.673 g), 1.6 mmol AgCl (0.229 g), 0.4 mmol NaCl (0.023 g), 1 mmol InCl_3_ (0.221 g) and 1 mmol BiCl_3_ (0.315 g) were dissolved in 1 mL dimethyl sulfoxide (DMSO) to form the Cs_2_Ag_0.8_Na_0.2_In_0.5_Bi_0.5_Cl_6_ slurry. After these steps, the paste was applied to form Cs_2_Ag_0.8_Na_0.2_In_0.5_Bi_0.5_Cl_6_ film. Wherein, the speed of the blade coater was set as 5 mm/s. The reason for using DMSO here was that a solvent with strong polarity and high boiling point was critical for the crystallization of LFHDPs, which was further beneficial for the synthesis of high-quality films^30^. Additionally, the chemical mixture that needs to be ground can be better dissolved in polar solvents. Especially for AgCl powder with poor solubility.

### Measurement and characterization

PL and PLQY measurements were performed by using the OmniFluo900 fluorescence spectrophotometer (Beijing Zhuolihanguang Instrument Co., Ltd, China). The PLQYs were measured at room temperature (RT) using a xenon lamp as excitation source coupled with an integrating sphere. Time-resolved PL experiments were conducted using the Edinburgh FLS1000 Instrument (Edinburgh Instruments Limited, UK). The XRD analysis was performed on a D/max 2200PC X-ray diffractometer (Nikkei Electric Co., Ltd, Japan), equipped with a 3 kW CuK_α_ ceramic X-ray tube. The experiment measurement 2 theta range is from 10 to 80°, with a scanning step of 0.04°. XPS measurements were carried out with an ESCALAB 250Xi instrument from the manufacturer (Temo Fisher Scientific, America). Samples were taken using a ZEISS GeminiSEM 300 scanning electron microscope (SEM) equipped with a Schottky Field Electron Emission Gun (Zeiss, Germany). Energy-dispersive spectroscopy (EDS) was used Oxford Xplore to evaluate the elemental ratios. The UV–visible absorption spectra were recorded using a Shimadzu UV-3600i Plus UV–vis absorption spectrophotometer (Shimadzu, Japan). TGA and DSC were measured by a STA 449/DSC 200 Simultaneous Thermal Analyzer (NETZSCH, Germany).

### Supplementary Information

Supplementary Information.

## Data Availability

The data that support the findings of this study are available from the corresponding author upon reasonable request.
