# Supplementary material for: Lead-free Cs2Ag1−xNaxIn1 − yBiyCl6 perovskite films with broad warm-yellow emission for lighting applications
Source: Sci Rep. 2024 Jun 26;14:14740. doi: 10.1038/s41598-024-65492-5 (PMC11208565; doi:10.1038/s41598-024-65492-5)
Supplement: Supplementary file 1 — Supplementary Information. [file 41598_2024_65492_MOESM1_ESM.docx]

***Supplementary Information***

**Lead-free Cs_2_Ag_1-x_Na_x_In_1-y_Bi_y_Cl_6_ perovskite films with broad warm-yellow emission for lighting applications**

Haiyan Wang^a^, Jin Chen^a,*^, Yu Sun^a^, Fengchao Wang^a,*^, Jing Yang^a^, Canyun Zhang^a^, Jinfang Kong^a^, Lan Li^a^

^1^ College of Sciences, Shanghai Institute of Technology, 100 Haiquan Road, Shanghai 201418, China

^*^ *Email*: [jinchenxl@sit.edu.cn](mailto:jinchenxl@sit.edu.cn) (Jin Chen); [fcwang@sit.edu.cn](mailto:fcwang@sit.edu.cn) (Fengchao Wang); *Tel*: 86-21-60873193

**Supporting Information Table of Contents:**

[**Supplementary Methods 2**](#_Toc167045089)

[**PLQY caculation 2**](#_Toc167045090)

[**TPRL analysis 2**](#_Toc167045091)

[**Crystal analysis 3**](#_Toc167045092)

[**Willamson-Hall plot analysis 3**](#_Toc167045093)

[**Supplementary Figures 5**](#_Toc167045094)

[**Supplementary Tables 5**](#_Toc167045095)

[**Reference 9**](#_Toc167045096)

# Supplementary Methods

## PLQY caculation

PLQY calculation was based on the Eq. (1)^1,2^

|  | (1) |
| --- | --- |

Where$A_{sample}$and$A_{reference}$are the absorption intensity of an integrating sphere with and without sample in a blank vessel, respectively, and $I_{sample}$is the integrated emission intensity of samples collected by the same integrating sphere. The fluorescence spectrometer we use comes with QY calibration files and attenuation plate calibration files. The QY test is divided into four steps, and the data obtained from the test is the data corrected for instrument responsiveness. Curve 0 and curve 1 are the direct and indirect measurement curves of the background sample. Curve 2 and curve 3 are direct and indirect measurement curves for Cs_2_Ag_1-x_Na_x_In_1-y_Bi_y_Cl_6_ film samples. PLQY can be calculated using the software provided by the fluorescence spectrometer. The testing steps are as follows.

**Step 1:** Place the background sample, the toggle on the right side of the integrating sphere is dialed to the vertical position and the excitation light hits the background sample.

**Step 2:** Place the background sample, the toggle on the right side of the integrating sphere is toggled to a horizontal position with the toggle facing the outside of the instrument, and the excitation light strikes the inner wall of the integrating sphere.

**Step 3:** Place the sample to be tested, the toggle on the right side of the integrating sphere is dialed back to the vertical position, and the excitation light strikes the sample to be tested.

**Step 4:** Place the sample to be tested, the toggle on the right side of the integrating sphere is dialed back to the horizontal position, the toggle is facing the outside of the instrument, and the excitation light strikes the inner wall of the integrating sphere.

## TPRL analysis

The value of *A_1_*, *A_2_*, *τ_1_*, *τ_2_* can be obtained through TRPL testing. The values of *τ_ave_*, *K_R_*, *K_NR_*, *K_R_/K_NR_* are calculated based on the TRPL data. The calculation formulas are as follows. *τ_aver_* refers to the average lifetime. *τ_1_* refers to the fitted short lifetime, and *τ_2_* refers to the fitted long lifetime. *A_1_* and *A_2_* are the weights of two exponential functions, *Φ* refers to *PLQY*, *K_R_*, and *K_NR_* refers to the radiation transition rate and the non-radiative transition rate, respectively.

| *τ_aver_ = (A_1_τ_1_^2^ + A_2_τ_2_^2^) / (A_1_τ_1_ + A_2_τ_2_)* | (2) |
| --- | --- |
| *τ_aver_ = (K_R_+K_NR_)^-1^* | (3) |
| *Φ = 1/(1+ K_NR_*/*K_R_)* | (4) |
| *τ_aver_ = Φ/K_R_* | (5) |

## Crystal analysis

In order to understand the growth changes of crystals, based on the XRD data, the FWHM and grain size (*D*) of the (220) crystal plane of the Cs_2_Ag_1-x_Na_x_In_1-y_Bi_y_Cl_6_ films were calculated by using Jade software. Based on the Scheler equation, *D* was also calculated. The corresponding data are shown in Supplementary Table 4 and Table 5. Scheler equation is listed in Eq. 6. Where $\beta$is the FWHM in radians. *K* is the shape factor. For cubic crystals, the *K* value is 0.943. $\lambda$=1.5406 Å is the wavelength of X-ray. *D* is the crystallite size and θ is the peak position in radians.

| $D=\frac{K\lambda}{\beta\cos\theta}$ | (6) |
| --- | --- |

## Willamson-Hall plot analysis

Crystallite size and micro strain from XRD data were calculated using the Willamson-Hall plot method. In XRD data, the broadening ($\beta_{T}$) of the peaks is due to the combined effect of crystallite size ($\beta_{D}$) and micro strain ($\beta_{\varepsilon}$). The equation is as follows.

| $\beta_{T}$=$\beta_{D}$+$\beta_{\varepsilon}$ | (7) |
| --- | --- |

Where$\beta_{T}$is the total broadening,$\beta_{D}$is the broadening due to crystallite size, and$\beta_{\varepsilon}$is the broadening due to strain.

Scherer equation is listed in Eq. 8. Eq. 8 can be transformed into Eq. 9. Where $\beta_{D}$is the FWHM in radians. *K* is the shape factor. For cubic crystals, the *K* value is 0.943. $\lambda$=1.5406 Å is the wavelength of X-ray. *D* is the crystallite size and$\theta$ is the peak position in radians. Eq. 10 illustrates how to calculate micro strain. Where $\varepsilon$is the strain.

Integrate Eq. 9, and Eq. 10 into Eq. 7, Eq. 11 can be obtained. Eq.11 can transform to Eq. 12.

In Eq. 12, considered$\beta_{T}\cos\theta$ as a function of $4\sin\theta$, it represents a straight line, where$\varepsilon$is the gradient (slope) of the line and $\frac{K\lambda}{D}$ is the y-intercept.

| $D=\frac{K\lambda}{\beta_{D}\cos\theta}$ | (8) |
| --- | --- |
| $\beta_{D}=\frac{K\lambda}{D\cos\theta}$ | (9) |
| $\beta_{\varepsilon}=4\varepsilon\tan\theta$ | (10) |
| $\beta_{T}$=$\frac{K\lambda}{D\cos\theta}$+$4\varepsilon\tan\theta$ | (11) |
| $\beta_{T}\cos\theta$=$\frac{K\lambda}{D}$+$4\varepsilon\sin\theta$ | (12) |

Based on Eq. 12, we plotted $4\sin\theta$ on x-axis and $\beta_{T}\cos\theta$ on y-axis. The slopes and y-intercepts through linear fitting were obtained. Then we calculated crystallite size from the y-intercept. Supplementary Figure 8 and Supplementary Figure 9 (a)-(f) are the Williamson-Hall plots of the Cs_2_Ag_1-x_Na_x_In_0.5_Bi_0.5_Cl_6_ and Cs_2_Ag_0.4_Na_0.6_In_1-y_Bi_y_Cl_6_ samples, respectively. Supplementary Table 6 and Supplementary Table 7 show $\varepsilon$and *D* of these samples.

# Supplementary Figures


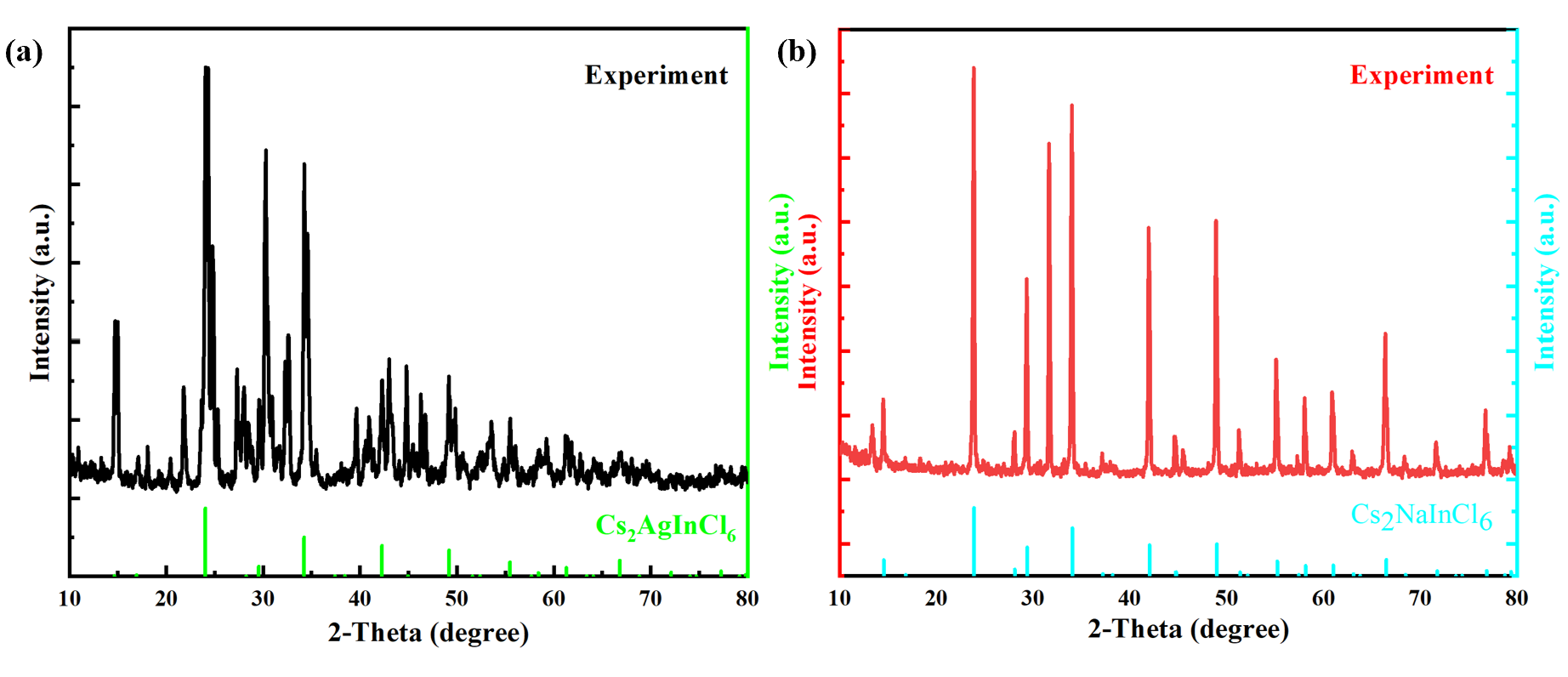


**Supplementary Figure 1.** (**a)** XRD pattern of experimental and standard Cs_2_AgInCl_6_. (**b)** XRD pattern of experimental and standard Cs_2_NaInCl_6_.


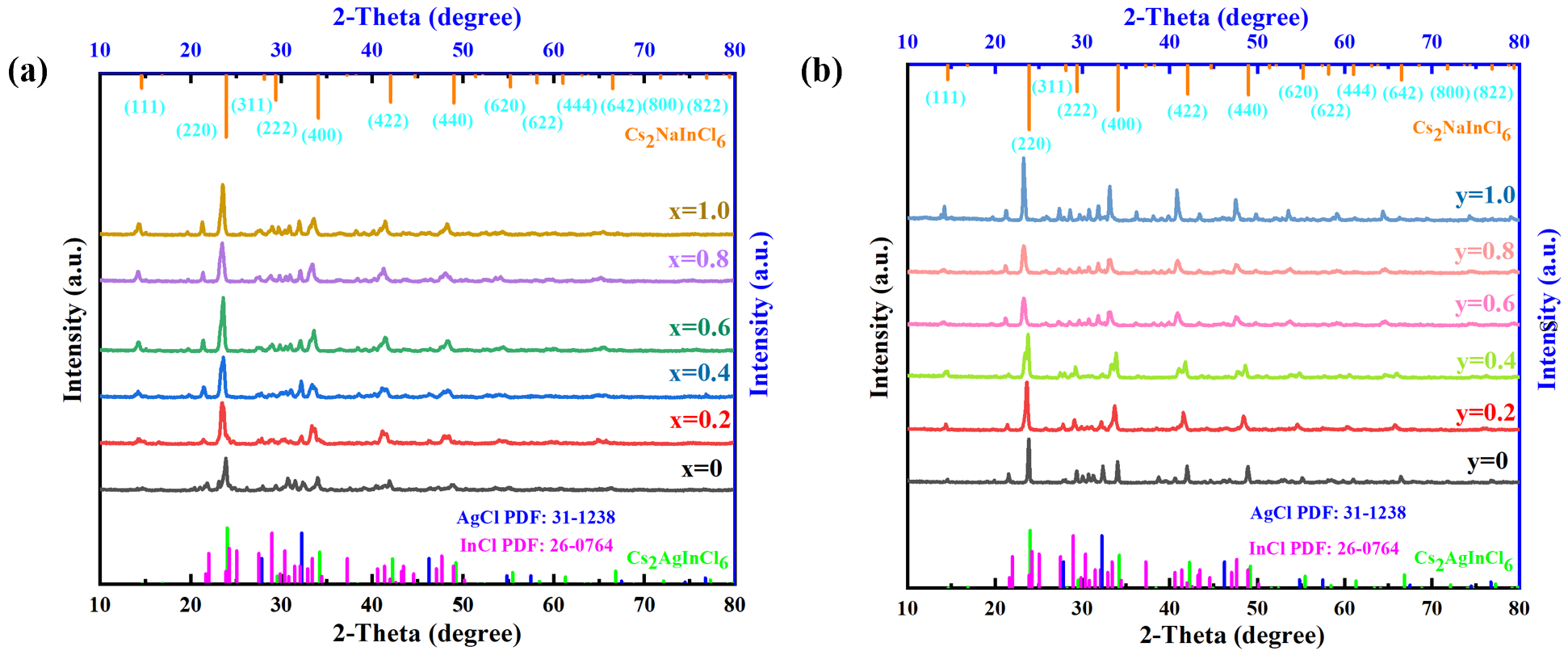


**Supplementary Figure 2.** XRD patterns of **(a)** Cs_2_Ag_1-x_Na_x_In_0.5_Bi_0.5_Cl_6_ samples. **(b)** Cs_2_Ag_0.4_Na_0.6_In_1-y_Bi_y_Cl_6_ samples.


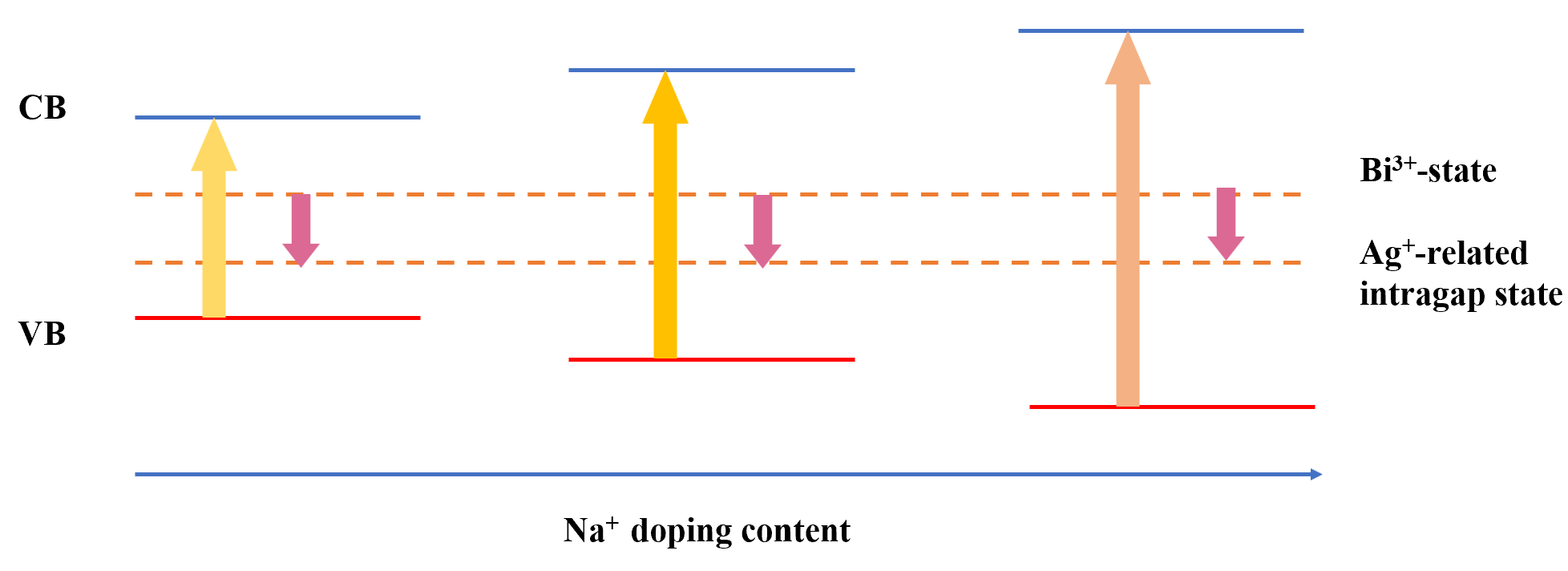


**Supplementary Figure 3.** The schematic depiction of the energy levels involved in the photophysics of CsAg_1-x_Na_x_In_0.5_Bi_0.5_Cl_6_ films.

**
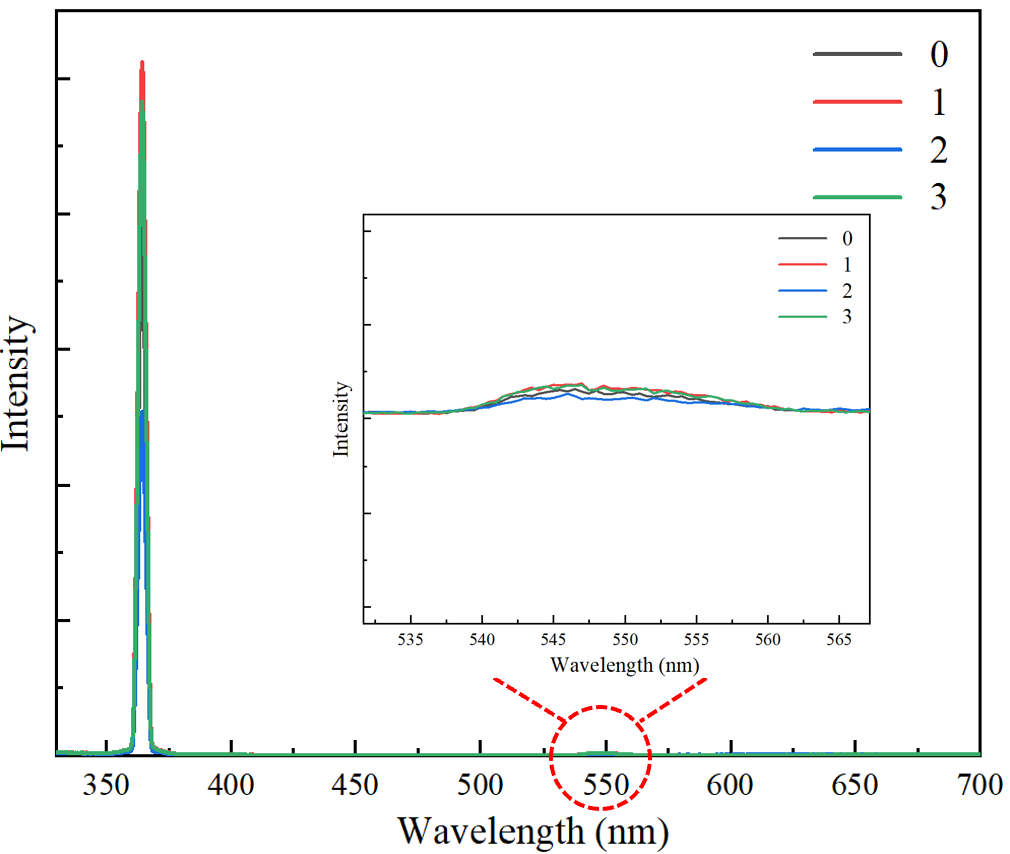
**

**Supplementary Figure 4.** PLQY test chart

**
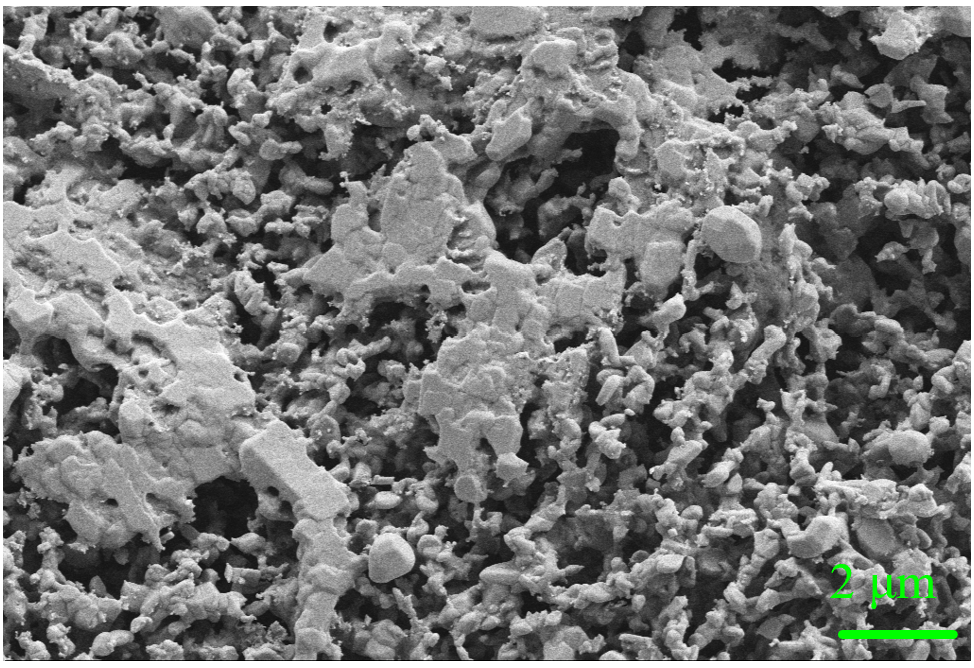
**

**Supplementary Figure 5.** SEM image of Cs_2_AgInCl_6_


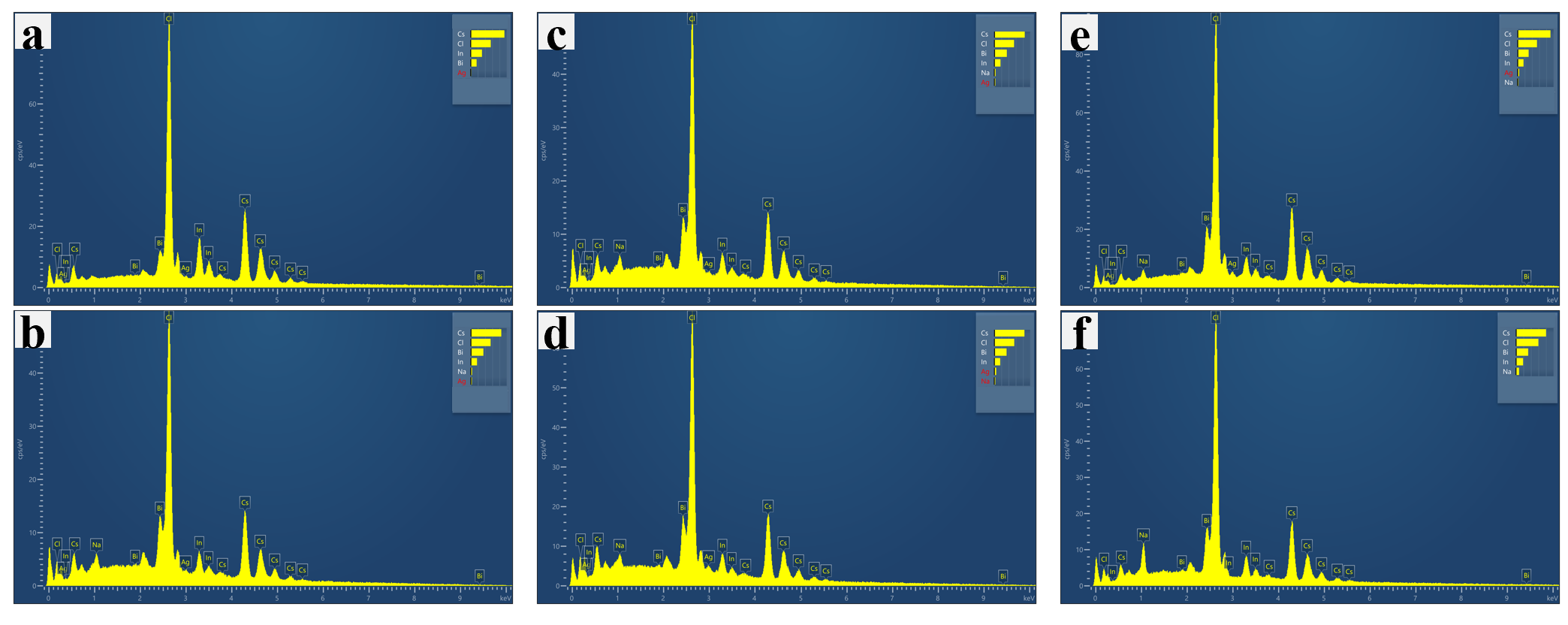


**Supplementary Figure 6.** EDS images of Cs_2_Ag_1-x_Na_x_In_0.5_Bi_0.5_Cl_6_ with different content of Na. **(a)** 0, **(b)** 0.2, **(c)** 0.4, **(d)** 0.6, **(e)** 0.8, and **(f)** 1.0, respectively.


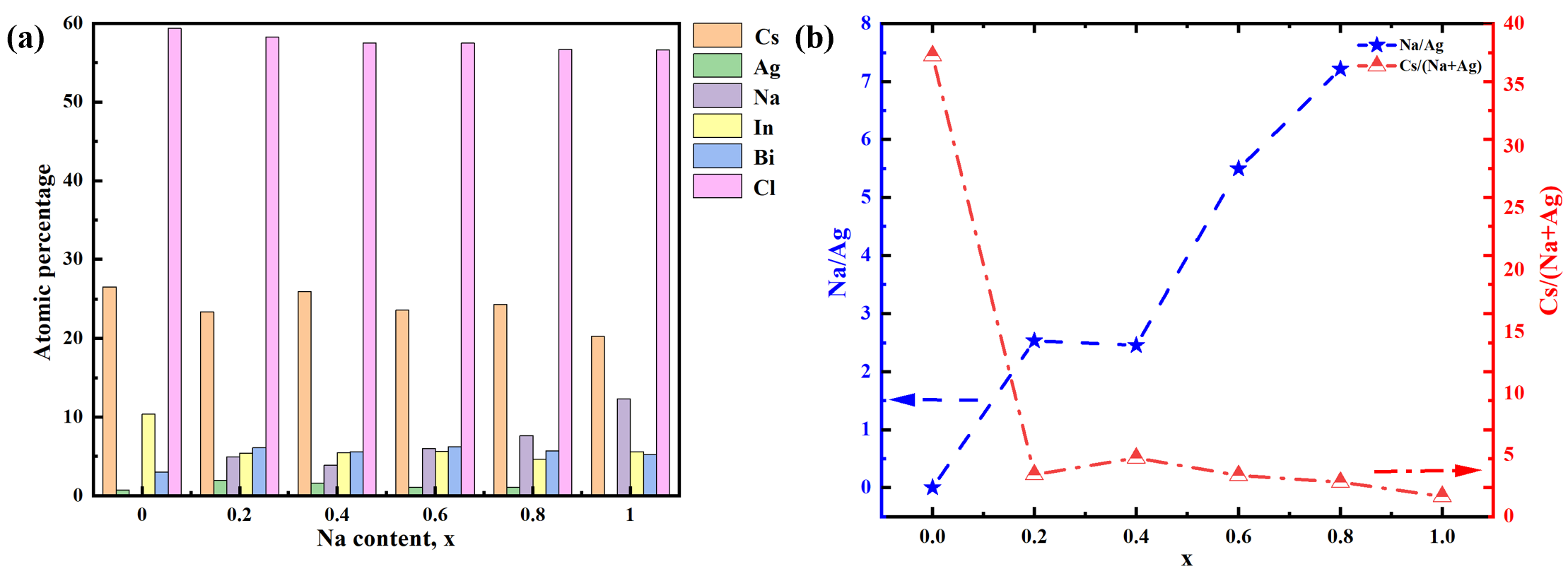


**Supplementary Figure 7.** **(a)** The atomic percentage of Cs_2_Ag_1-x_Na_x_In_0.5_Bi_0.5_Cl_6_ samples was measured by EDS. **(b)** The Na/Ag and Cs/(Na+Ag) ratio with the change of the value of x according to the data listed in Supplementary Table 1.

**
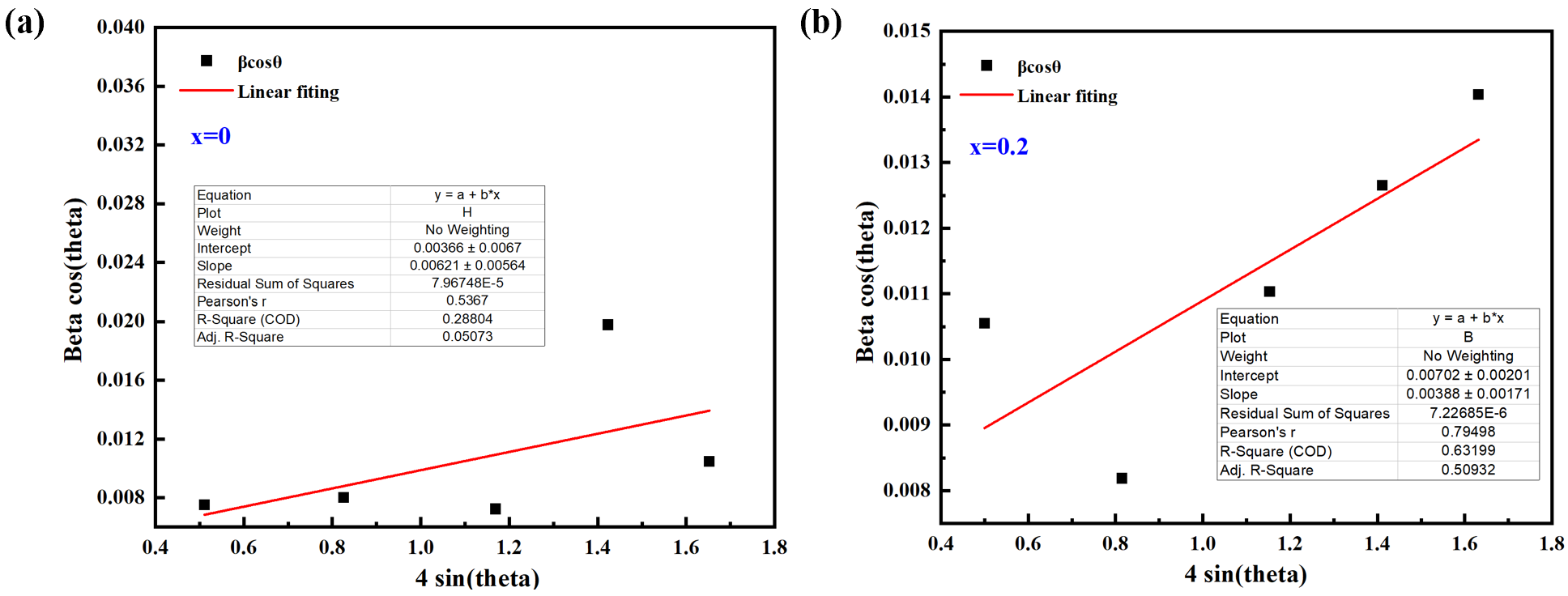
**

**
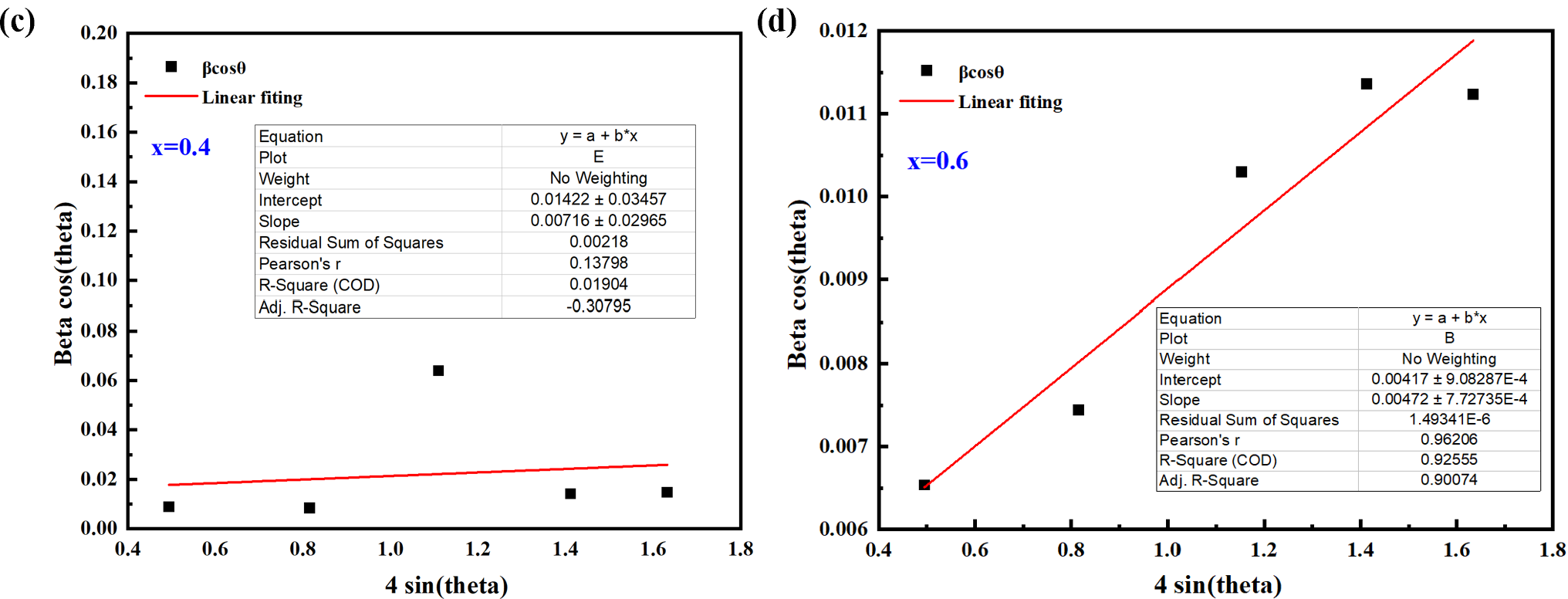
**

**
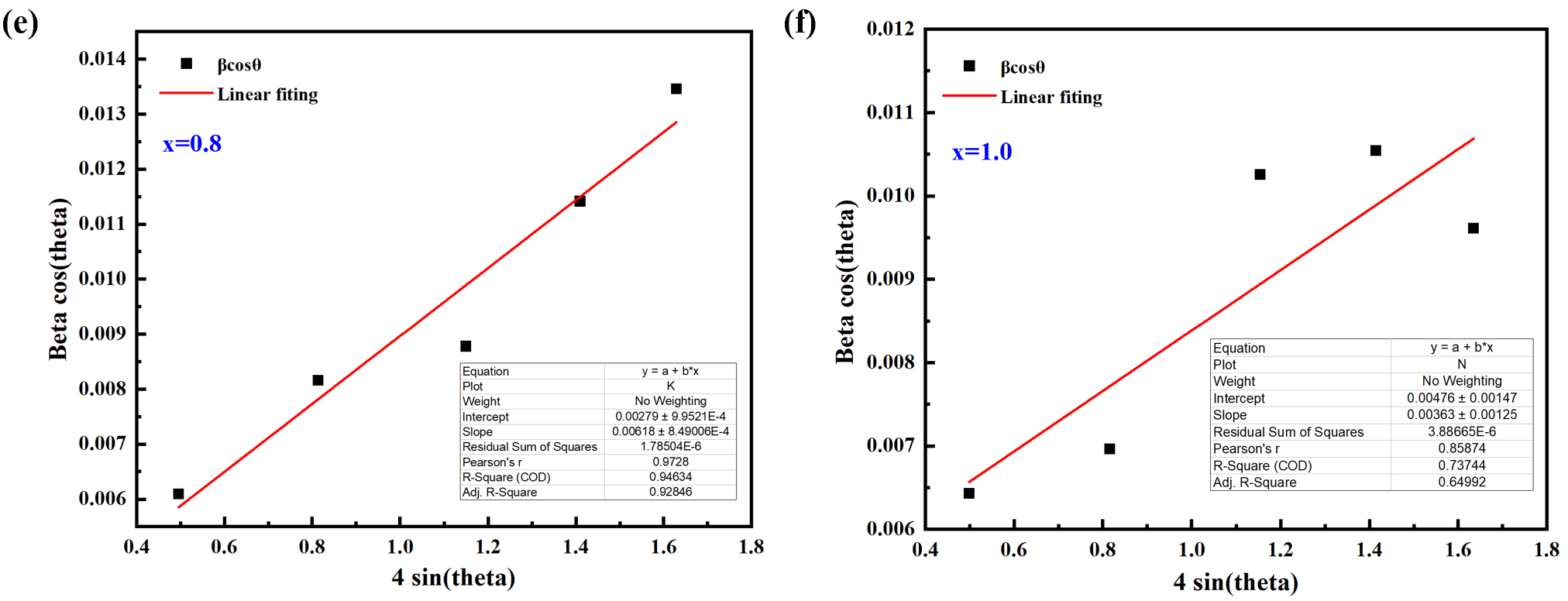
**

**Supplementary Figure 8.** The linear fitting results of Cs_2_Ag_1-x_Na_x_In_0.5_Bi_0.5_Cl_6_ samples. **(a)** x=0, **(b)** x=0.2, **(c)** x=0.4, **(d)** x=0.6, **(e)** x=0.8, **(f)** x=1.0.


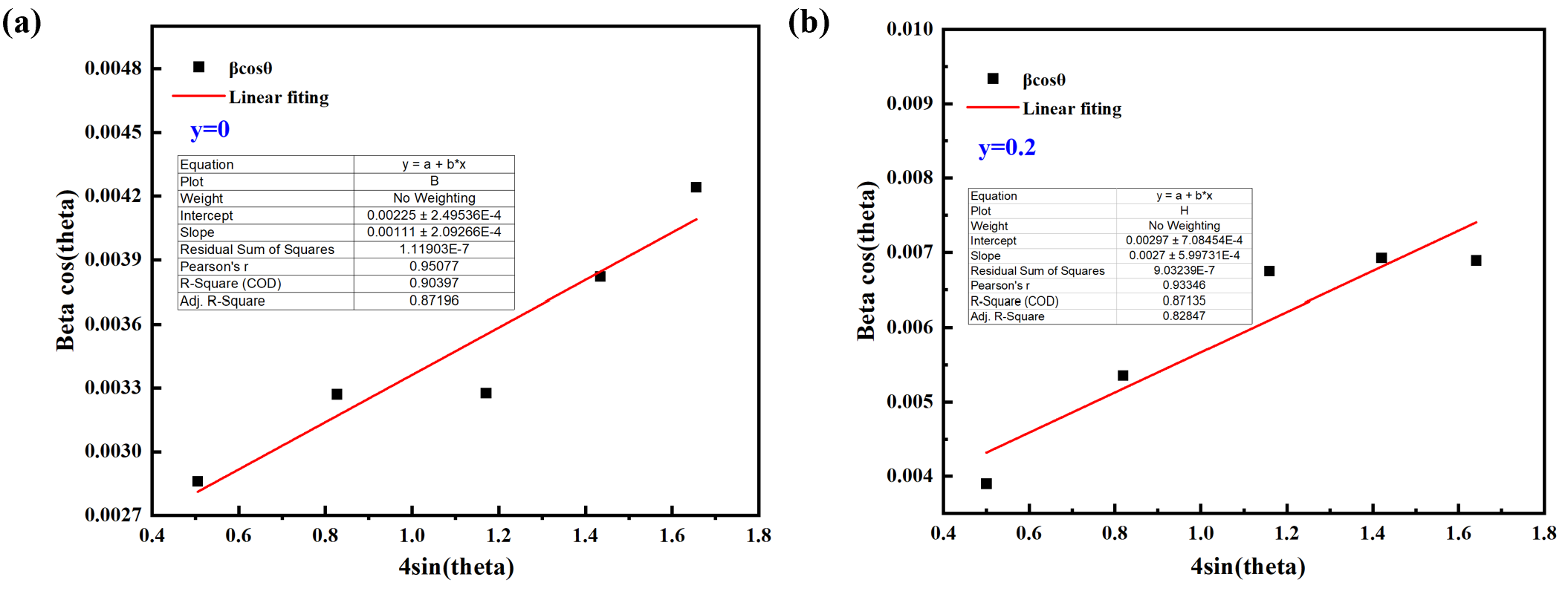


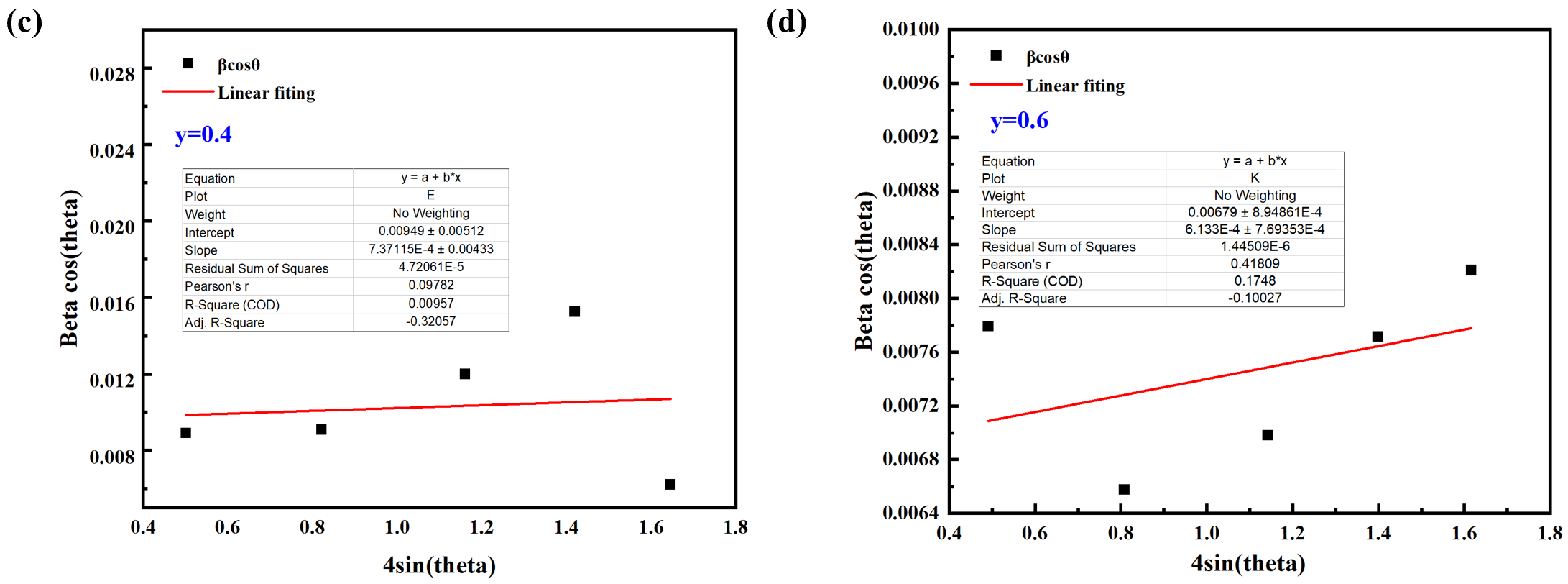


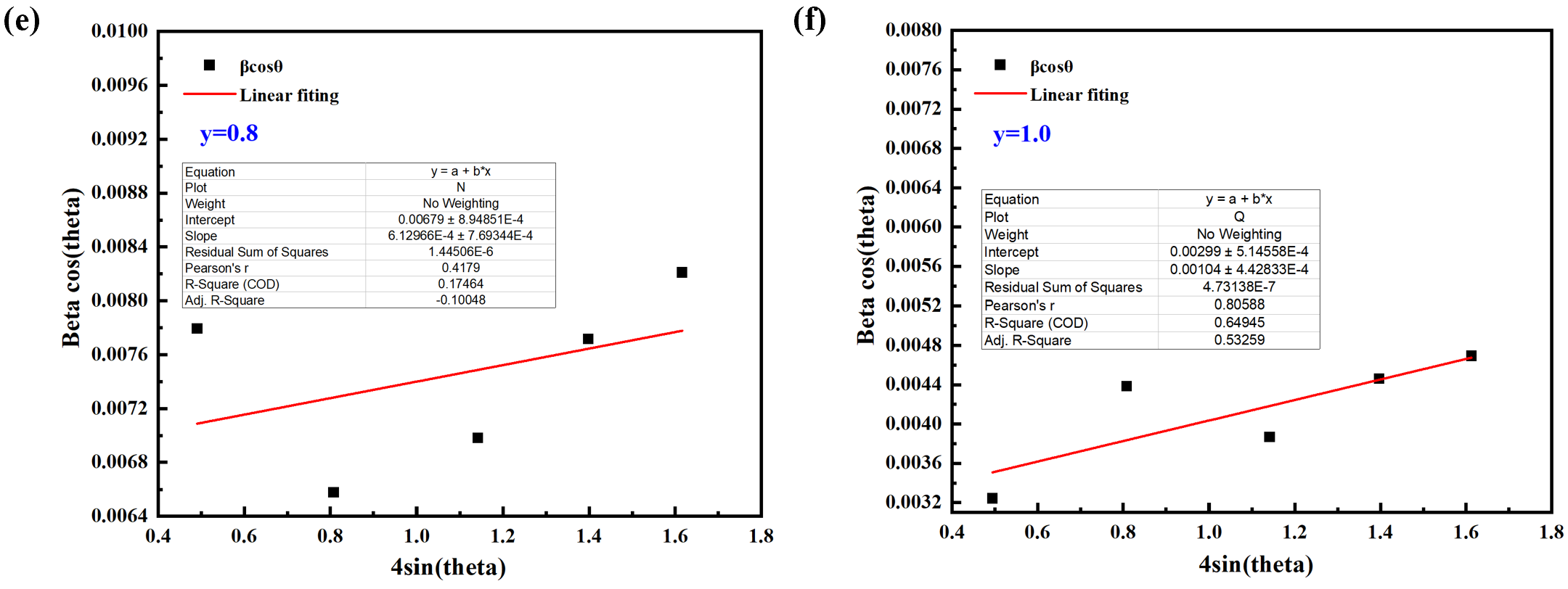


**Supplementary Figure 9.** The linear fitting results of Cs_2_Ag_0.4_Na_0.6_In_1-y_Bi_y_Cl_6_ samples. **(a)** y=0, **(b)** y=0.2, **(c)** y=0.4, **(d)** y=0.6, **(e)** y=0.8, **(f)** y=1.0.


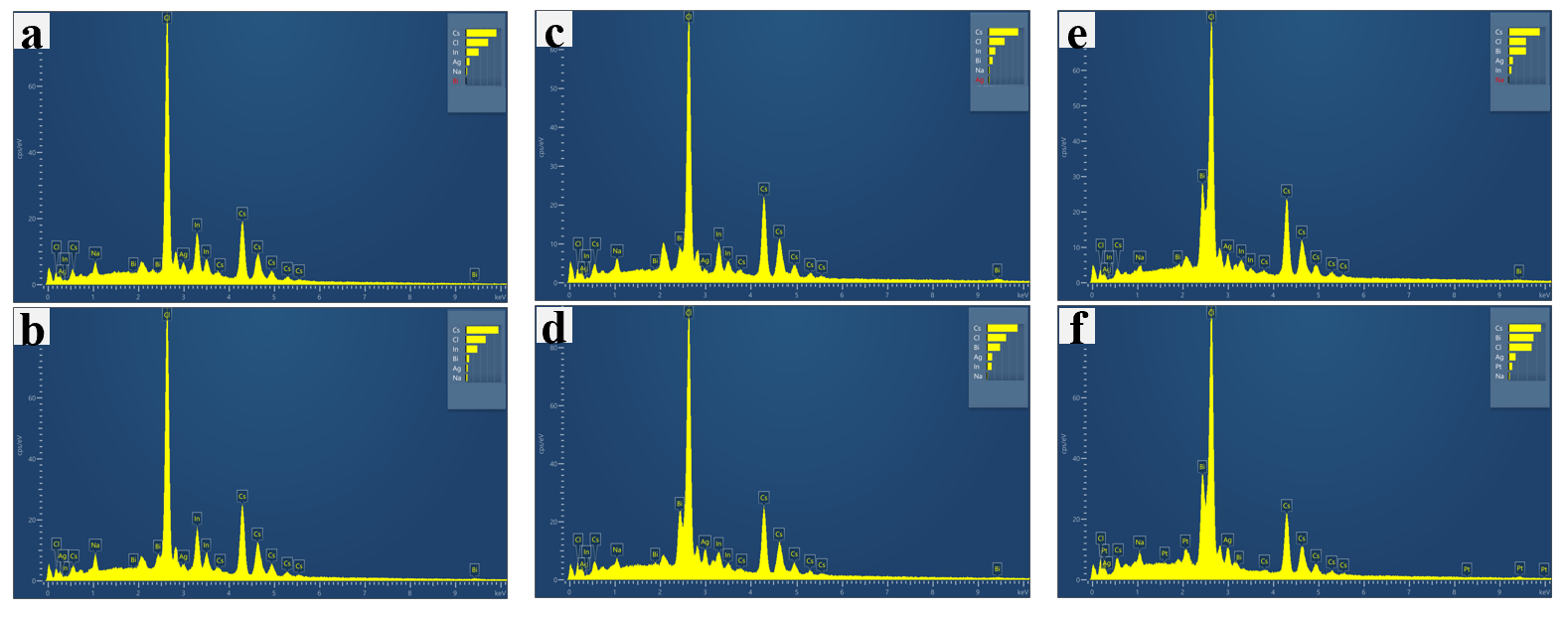


**Supplementary Figure 10.** EDS images of Cs_2_Ag_0.4_Na_0.6_In_1-y_Bi_y_Cl_6_ with different content of Bi. **(a)** 0, **(b)** 0.2, **(c)** 0.4, **(d)** 0.6, **(e)** 0.8, and **(f)** 1.0, respectively.


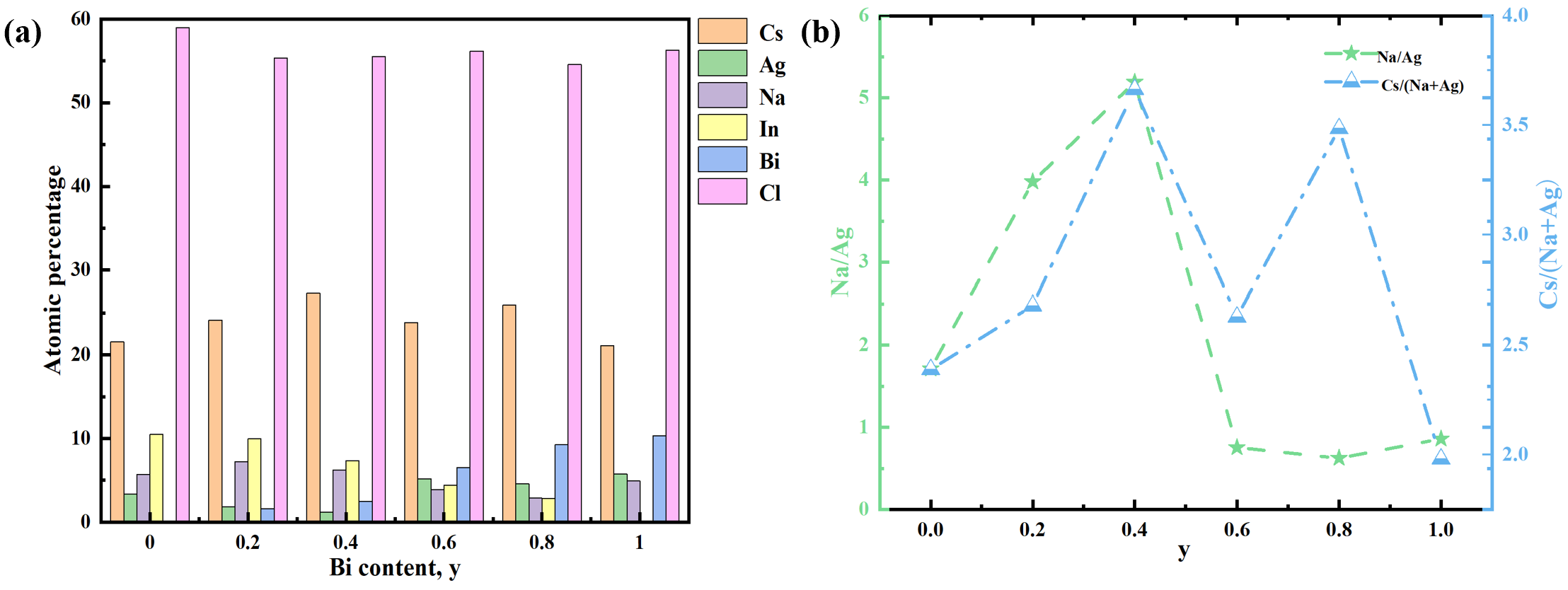


**Supplementary Figure 11.** **(a)** The atomic percentage of Cs_2_Ag_0.4_Na_0.6_In_1-y_Bi_y_Cl_6_ samples was measured by EDS. **(b)** The Na/Ag and Cs/(Na+Ag) ratio with the change of the value of y according to the data listed in Supplementary Table 2.


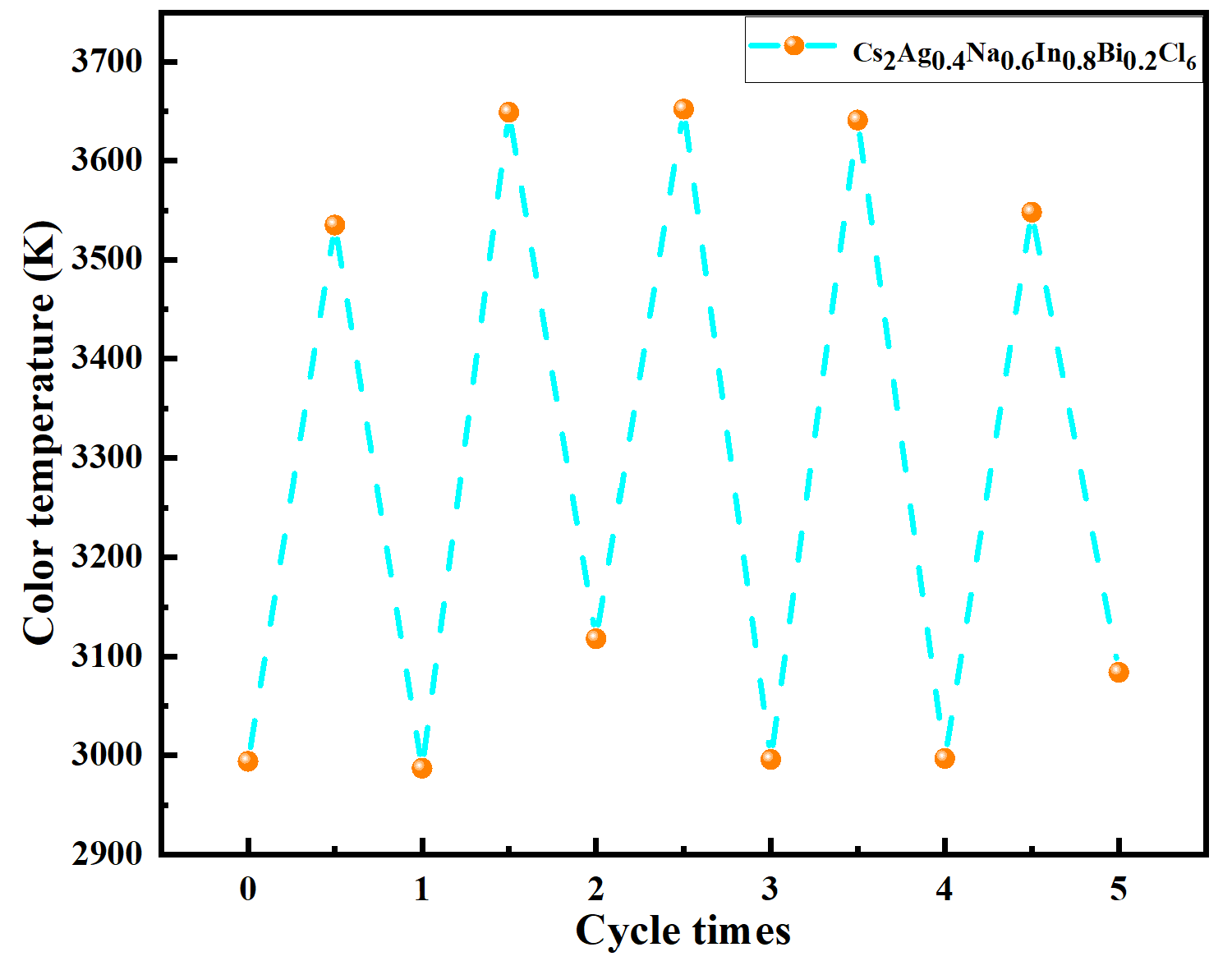


**Supplementary Figure 12.** The color temperature of the Cs_2_Ag_0.6_Na_0.4_In_0.8_Bi_0.2_Cl_6_ sample at 20−100℃.


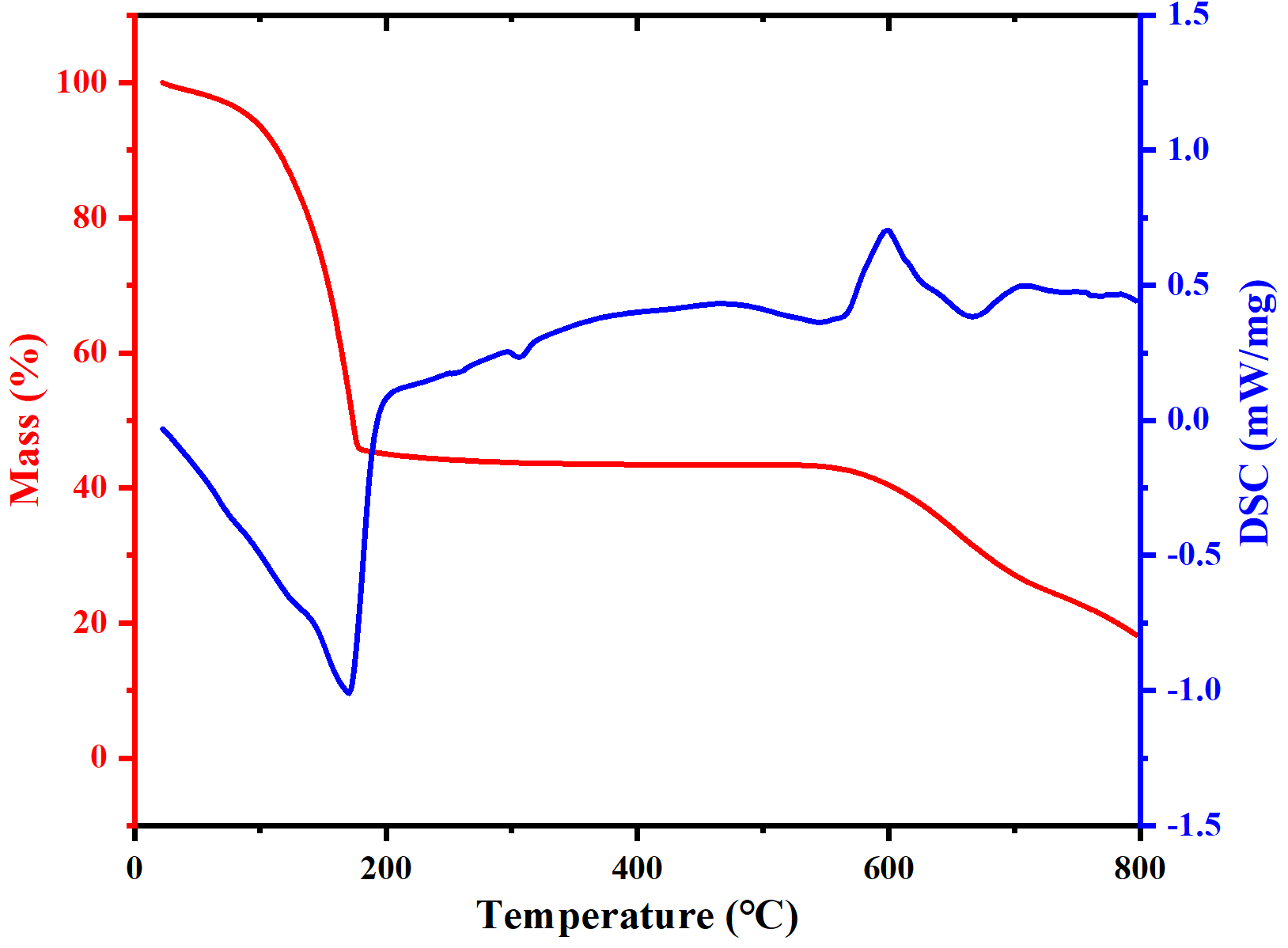


**Supplementary Figure 13.** Thermogravimetric analysis (TGA) and differential scanning calorimeter (DSC) analysis of the Cs_2_Ag_0.6_Na_0.4_In_0.8_Bi_0.2_Cl_6_ sample.


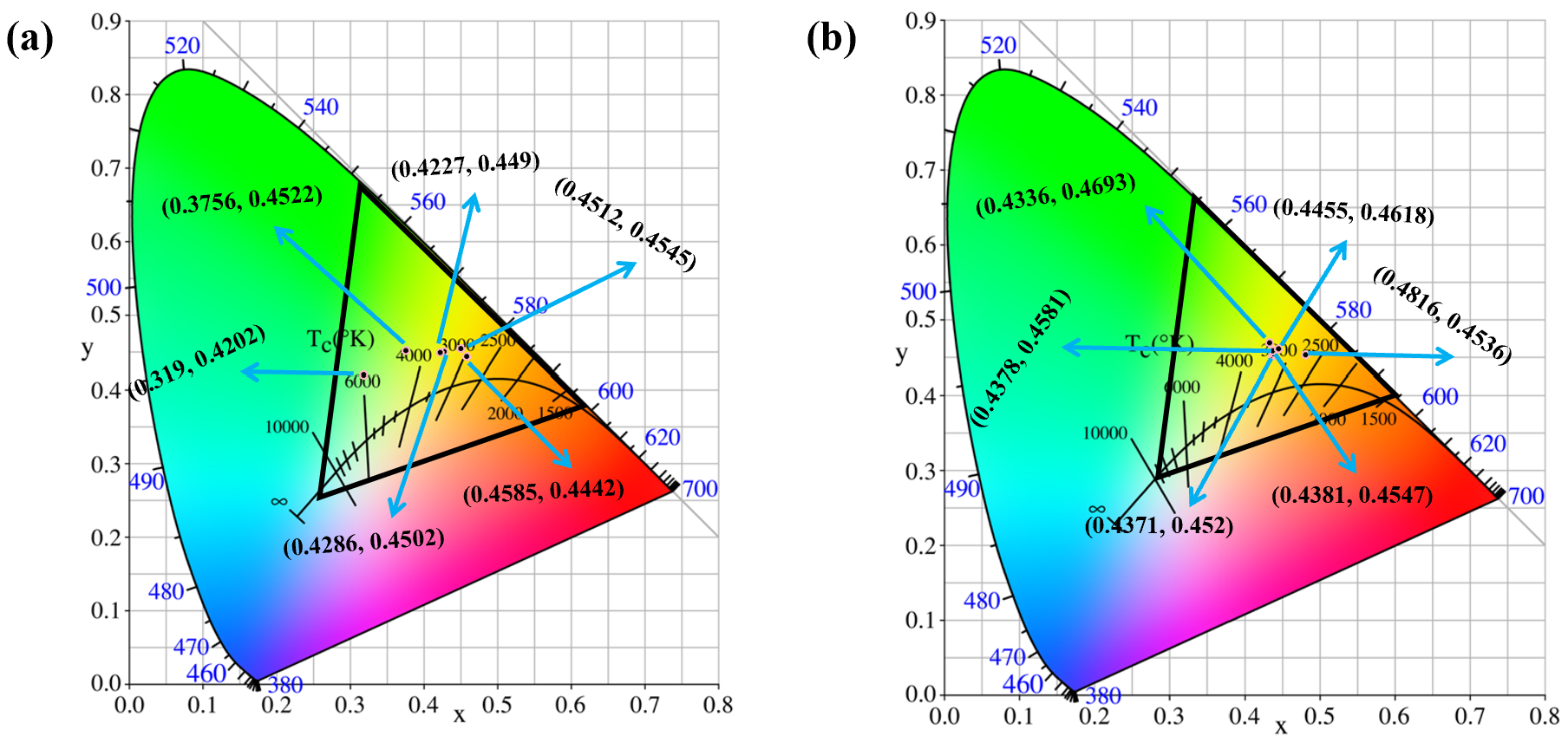


**Supplementary Figure 14.** CIE color coordinates plotted on the CIE color space chromaticity chart. **(a)** Cs_2_Ag_1-x_Na_x_In_0.5_Bi_0.5_Cl_6_ samples. **(b)** Cs_2_Ag_0.4_Na_0.6_In_1-y_Bi_y_Cl_6_ samples.


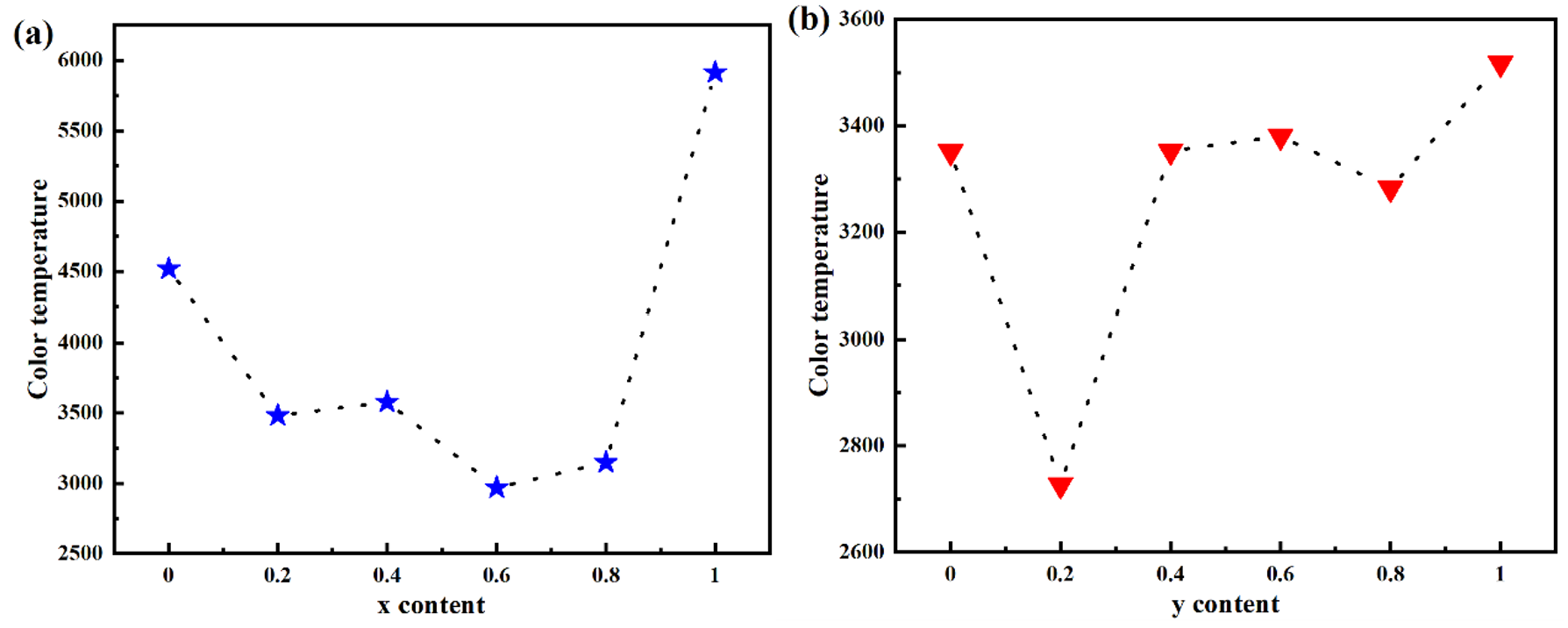


**Supplementary Figure 15.** The color temperature of **(a)** Cs_2_Ag_1-x_Na_x_In_0.5_Bi_0.5_Cl_6_ samples. **(b)** Cs_2_Ag_0.4_Na_0.6_In_1-y_Bi_y_Cl_6_ samples.


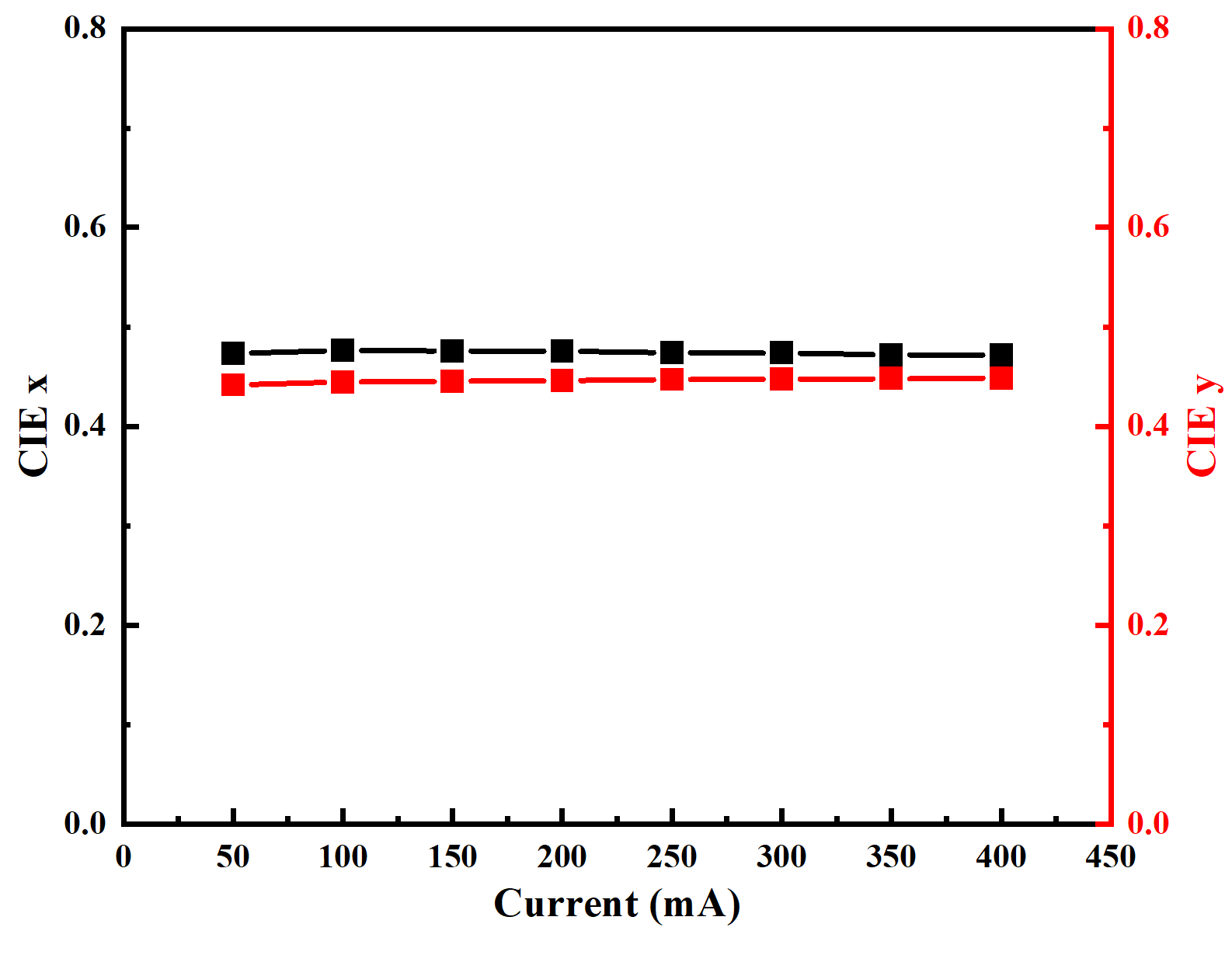


**Supplementary Figure 16.** CIE color coordinates for the Cs_2_Ag_0.4_Na_0.6_In_0.8_Bi_0.2_Cl_6_ LED at driving currents from 50 to 400 mA.

# Supplementary Tables

**Supplementary Table 1.** The details of *τ_1_*, *τ_2_*, *τ_ave_*, PLQY, *K_R_*, *K_NR_*, and *K_R_/K_NR_* for Cs_2_Ag_1-x_Na_x_In_0.5_Bi_0.5_Cl_6_ samples with the change of x.

| samples | *τ_1_*  (μs) | *A_1_*  (%) | *τ_2_*  (μs) | *A_2_*  (%) | *τ_ave_*  (μs) | *PLQY*  (%) | *K_R_*  (10^6s^-1^) | *K_NR_* (10^6s^-1^) | *K_R_/K_NR_* |
| --- | --- | --- | --- | --- | --- | --- | --- | --- | --- |
| x=0 | 0.47 | 15.95 | 4.50 | 84.05 | 3.86 | 10.39 | 0.027 | 0.232 | 0.116 |
| x=0.2 | 0.44 | 32.11 | 3.06 | 67.89 | 2.22 | 12.79 | 0.058 | 0.392 | 0.148 |
| x=0.4 | 0.38 | 21.47 | 3.73 | 78.53 | 3.01 | 21.26 | 0.071 | 0.261 | 0.272 |
| x=0.6 | 0.44 | 17.07 | 4.23 | 82.93 | 3.58 | 21.6 | 0.060 | 0.219 | 0.274 |
| x=0.8 | 0.57 | 13.20 | 4.73 | 86.80 | 4.18 | 10.67 | 0.026 | 0.231 | 0.122 |
| x=1.0 | 0.51 | 11.66 | 4.61 | 88.34 | 4.13 | / | / | / | / |

**Supplementary Table 2.** The calculated elements ratio of Cs_2_Ag_1-x_Na_x_In_0.5_Bi_0.5_Cl_6_ samples.

|  | Na/Ag | Bi/In | Cs/(Na+Ag) | Cs/(In+Bi) | Cl/Cs |
| --- | --- | --- | --- | --- | --- |
| x=0 | 0 | 0.29 | 37.39 | 1.98 | 2.24 |
| x=0.2 | 2.54 | 1.13 | 3.41 | 2.03 | 2.49 |
| x=0.4 | 2.46 | 1.02 | 4.76 | 2.35 | 2.21 |
| x=0.6 | 5.50 | 1.10 | 3.33 | 1.99 | 2.44 |
| x=0.8 | 7.22 | 1.23 | 2.79 | 2.36 | 2.33 |
| x=1.0 | / | 0.94 | 1.64 | 1.87 | 2.80 |

**Supplementary Table 3.** The details of *τ_1_*, *τ*_2_, *τ*_ave_, *PLQY*, *K_R_*, *K_NR_*, and *K_R_*/*K_NR_* for Cs_2_Ag_0.4_Na_0.6_In_1-y_Bi_y_Cl_6_ samples with the change of y.

| Samples | *τ_1_*  (μs) | *A_1_*  (%) | *τ_2_*  (μs) | *A_2_*  (%) | *τ_ave_*  (μs) | *PLQY*  (%) | *K_R_*  (10^6s^-1^) | *K_NR_* (10^6s^-1^) | *K_R_/K_NR_* |
| --- | --- | --- | --- | --- | --- | --- | --- | --- | --- |
| y=0 | 0.28 | 19.71 | 4.88 | 80.29 | 3.97 | 14.37 | 0.036 | 0.216 | 0.167 |
| y=0.2 | 0.59 | 13.89 | 3.81 | 86.11 | 3.36 | 66.38 | 0.198 | 0.01 | 19.8 |
| y=0.4 | 0.39 | 20.33 | 3.64 | 79.67 | 2.98 | 23.95 | 0.08 | 0.256 | 0.313 |
| y=0.6 | 0.25 | 39.14 | 3.27 | 60.86 | 2.09 | 21.39 | 0.102 | 0.376 | 0.271 |
| y=0.8 | 0.38 | 27.12 | 3.30 | 72.88 | 2.51 | 20.35 | 0.081 | 0.317 | 0.256 |
| y=1.0 | 2.76 | 22.12 | 3.91 | 77.88 | 3.66 | 20.39 | 0.056 | 0.217 | 0.258 |

**Supplementary Table 4.** Crystal data of Cs_2_Ag_1-x_Na_x_In_0.5_Bi_0.5_Cl_6_ samples on (220) plane.

|  | *θ* | *d* (nm) | FWHM | lattice | Average lattice | *D* (Å) |
| --- | --- | --- | --- | --- | --- | --- |
| x=0 | 23.88 | 3.7232 | 0.434 | 10.5308 | 10.2225 | 196 |
| x=0.2 | 23.48 | 3.7857 | 0.478 | 10.7076 | 10.6167 | 178 |
| x=0.4 | 23.6 | 3.7667 | 0.435 | 10.6539 | 10.9897 | 195 |
| x=0.6 | 23.6 | 3.7667 | 0.362 | 12.4928 | 11.7117 | 235 |
| x=0.8 | 23.48 | 3.7857 | 0.414 | 10.7076 | 10.7076 | 205 |
| x=1.0 | 23.52 | 3.7794 | 0.353 | 13.0959 | 10.6896 | 241 |

**Supplementary Table 5.** Crystal data of Cs_2_Ag_0.4_Na_0.6_In_1-y_Bi_y_Cl_6_ samples on (220) plane.

|  | *θ* | *d* (nm) | FWHM | lattice | Average lattice | *D* (Å) |
| --- | --- | --- | --- | --- | --- | --- |
| y=0 | 23.87 | 3.7250 | 0.18 | 10.5360 | 10.5360 | 423 |
| y=0.2 | 24.0 | 3.7082 | 0.233 | 10.4884 | 10.4884 | 365 |
| y=0.4 | 23.68 | 3.7542 | 0.289 | 10.6184 | 10.3804 | 294 |
| y=0.6 | 23.80 | 3.7355 | 0.351 | 10.5656 | 9.5782 | 242 |
| y=0.8 | 23.28 | 3.8178 | 0.368 | 10.7983 | 19.1363 | 231 |
| y=1.0 | 23.28 | 3.8178 | 0.217 | 10.7983 | 9.8156 | 392 |

STEs are widely present in metal halide perovskites. Broadband emission mainly origins from the STEs with Jahn-Teller-like octahedral distortions^3-5^.

In a STE process, the emissive energy (*E_PL_*) can be described by the follow equation, where *E_g_* is the band gap energy, *E_b_* is the exciton binding energy, E_st_ is the self-tapping energy, and E_d_ is the lattice deformation energy, which is directly relevant to the degree of lattice distortion. Each emissive STE can have an appropriate degree of lattice distortion^6^. *E_g_* and *E_b_* are constant. Therefore, *E_PL_* is determined by *E_st_* and *E_d_*^7^. Since there are different STE structures in each perovskite, *E_st_* and *E_d_* are different^3-5,7^. A series of lattice paraments of Cs_2_Ag_1-x_Na_x_In_1-y_Bi_y_Cl_6_ films have been listed in the Supplementary Table 6 and Table 7 of the supporting information. The change in lattice behavior is significant.

| $E_{PL}=E_{g}-E_{b}-E{}_{st}-E_{d}$ | (13) |
| --- | --- |

From Supplementary Table 6, it can be noticed that the interplanar spacing (*d*) value does not change significantly and remains around 3.7 nm. However, *d* value tends to become bigger in Cs_2_Ag_0.4_Na_0.6_In_1-y_Bi_y_Cl_6_ samples with the increase of Bi^3+^ doping content in Supplementary Table 7. This is related to lattice expansion caused by doping. Using *D* as an example, Na^+^ has a smaller radius than Ag^+^. When Na^+^ ions replace some Ag^+^ ions, except for the sample with x=0.8, the *D* value increases for the sample from x=0.2 to x=1.0. It was found that a decrease in (220) peak intensity from the XRD pattern of the x=0.8 sample could originate from the Na^+^ and Ag^+^ disorder in Cs_2_Ag_0.2_Na_0.8_In_1-y_Bi_y_Cl_6_ samples. For Cs_2_Ag_0.4_Na_0.6_In_1-y_Bi_y_Cl_6_ samples, except for the sample with y=1.0, the *D* value decreases with the increase of Bi^3+^ doping. Additionally, due to the difference in ion sizes before and after doping, grain expansion or contraction, external and internal stresses, and impurities may affect *D* together. Combining Eq.13, a conclusion can be drawn that the change in optical bandgap of the materials is relevant to the change in lattice behavior, mainly by lattice deformation.

The data in Supplementary Table 7 and Supplementary Table 8 are obtained based on the fitting results in Supplementary Figure 8 and Supplementary Figure 9, respectively. There lies some changes in average grain size (*D*) with the Na^+^ and Bi^3+^ doping content.

**Supplementary Table 6.** The calculated data of Cs_2_Ag_1-x_Na_x_In_0.5_Bi_0.5_Cl_6_ samples.

|  | *ε* | *D* (Å) |
| --- | --- | --- |
| x=0 | 0.00621 | 396.94 |
| x=0.2 | 0.00388 | 206.95 |
| x=0.4 | 0.00716 | 102.17 |
| x=0.6 | 0.00472 | 348.39 |
| x=0.8 | 0.00618 | 520.71 |
| x=1.0 | 0.00363 | 305.21 |

**Supplementary Table 7.** The calculated data of Cs_2_Ag_0.4_Na_0.6_In_1-y_Bi_y_Cl_6_ samples.

|  | *ε* | *D* (Å) |
| --- | --- | --- |
| y=0 | 0.00111 | 348.39 |
| y=0.2 | 0.00270 | 645.68 |
| y=0.4 | 0.00074 | 489.15 |
| y=0.6 | 0.00061 | 153.09 |
| y=0.8 | 0.00061 | 213.96 |
| y=1.0 | 0.00104 | 485.88 |

**Supplementary Table 8.** The calculated elements ratio of Cs_2_Ag_0.4_Na_0.6_In_1-y_Bi_y_Cl_6_ samples.

|  | Na/Ag | Bi/In | Cs/(Na+Ag) | Cs/(In+Bi) | Cl/Cs |
| --- | --- | --- | --- | --- | --- |
| y=0 | 1.71 | 0 | 2.39 | 2.05 | 2.74 |
| y=0.2 | 3.98 | 0.16 | 2.68 | 2.10 | 2.29 |
| y=0.4 | 5.19 | 0.34 | 3.66 | 2.78 | 2.04 |
| y=0.6 | 0.75 | 1.50 | 2.63 | 2.19 | 2.35 |
| y=0.8 | 0.62 | 3.25 | 3.49 | 2.14 | 2.10 |
| y=1.0 | 0.86 | / | 1.98 | 2.04 | 2.67 |

**Supplementary Table 9.** The different values of CRI for tunable CCT of Cs_2_Ag_1-x_Na_x_In_1-y_Bi_y_Cl_6_ LEDs.

|  | CCT | CRI |
| --- | --- | --- |
| x=0 | 4519 | 73.1 |
| x=0.2 | 3482 | 83.4 |
| x=0.4 | 3577 | 83.2 |
| x=0.6 | 2969 | 87.8 |
| x=0.8 | 3148 | 84.2 |
| x=1.0 | 5913 | 69.5 |
| y=0 | 3351 | 82.2 |
| y=0.2 | 2727 | 83.9 |
| y=0.4 | 3352 | 83.1 |
| y=0.6 | 3380 | 81.7 |
| y=0.8 | 3282 | 81.3 |
| y=1.0 | 3517 | 76.3 |

## Reference

1 Zhou, C. et al. Luminescent zero-dimensional organic metal halide hybrids with near-unity quantum efficiency. *Chem. Sci.* **9**, 586-593 (2018).

2 Luo, J. et al. Efficient and stable emission of warm-white light from lead-free halide double perovskites. *Nature* **563**, 541-545 (2018).

3 Manna, D., Das, T. K. & Yella, A. Tunable and stable white light emission in Bi^3+^-alloyed Cs_2_AgInCl_6_ double perovskite nanocrystals. *Chem. Mater.* **31**, 10063-10070 (2019).

4 Wang, X. et al*.* Atomistic mechanism of broadband emission in metal halide perovskites. *J. Phys. Chem. Lett.* **10**, 501-506 (2019).

5 Li, Q. et al. Excitation-dependent emission color tuning of 0D Cs_2_InBr_5_·H_2_O at high pressure. *Adv. Funct. Materials* **31**, 2104923 (2021).

6 Zhou, B. et al. Efficient white photoluminescence from self-trapped excitons in Sb^3+^/Bi^3+^-codoped Cs_2_NaInCl_6_ double perovskites with tunable dual-emission. *ACS Energy Lett* **6**, 3343-3351 (2021).

7 Li, S., Luo, J., Liu, J. & Tang, J. Self-trapped excitons in all-inorganic halide perovskites: fundamentals, status, and potential applications. *J. Phys. Chem. Lett.* **10**, 1999-2007 (2019).
